# Liquorice Extract and 18β-Glycyrrhetinic Acid Protect Against Experimental Pyrrolizidine Alkaloid-Induced Hepatotoxicity in Rats Through Inhibiting Cytochrome P450-Mediated Metabolic Activation

**DOI:** 10.3389/fphar.2022.850859

**Published:** 2022-03-16

**Authors:** Zhangting Wang, Jiang Ma, Sheng Yao, Yisheng He, Kai-Kei Miu, Qingsu Xia, Peter P. Fu, Yang Ye, Ge Lin

**Affiliations:** ^1^ School of Biomedical Sciences, Faculty of Medicine, The Chinese University of Hong Kong, Hong Kong SAR, China; ^2^ State Key Laboratory of Drug Research and Natural Products Chemistry Department, Shanghai Institute of Materia Medica, Chinese Academy of Sciences, Shanghai, China; ^3^ National Center for Toxicological Research, U.S. Food and Drug Administration, Jefferson, AR, United States

**Keywords:** 18β-glycyrrhetinic acid, pyrrolizidine alkaloid, cytochrome P450, metabolic activation, competitive inhibition

## Abstract

Misuse of pyrrolizidine alkaloid (PA)-containing plants or consumption of PA-contaminated foodstuffs causes numerous poisoning cases in humans yearly, while effective therapeutic strategies are still limited. PA-induced liver injury was initiated by cytochrome P450 (CYP)-mediated metabolic activation and subsequent formation of adducts with cellular proteins. Liquorice, a hepato-protective herbal medicine, is commonly used concurrently with PA-containing herbs in many compound traditional Chinese medicine formulas, and no PA-poisoning cases have been reported with this combination. The present study aimed to investigate hepato-protective effects of liquorice aqueous extract (EX) and 18β-glycyrrhetinic acid (GA, the primary bioactive constituent of liquorice) against PA-induced hepatotoxicity and the underlying mechanism. Histopathological and biochemical analysis demonstrated that both single- and multiple-treatment of EX (500 mg/kg) or GA (50 mg/kg) significantly attenuated liver damage caused by retrorsine (RTS, a representative hepatotoxic PA). The formation of pyrrole-protein adducts was significantly reduced by single- (30.3% reduction in liver; 50.8% reduction in plasma) and multiple- (32.5% reduction in liver; 56.5% reduction in plasma) treatment of GA in rats. Single- and multiple-treatment of EX also decreased the formation of pyrrole-protein adducts, with 30.2 and 31.1% reduction in rat liver and 51.8 and 53.1% reduction in rat plasma, respectively. In addition, *in vitro* metabolism assay with rat liver microsomes demonstrated that GA reduced the formation of metabolic activation-derived pyrrole-glutathione conjugate in a dose-dependent manner with the estimated IC_50_ value of 5.07 µM. Further mechanism study showed that GA inhibited activities of CYPs, especially CYP3A1, the major CYP isoform responsible for the metabolic activation of RTS in rats. Enzymatic kinetic study revealed a competitive inhibition of rat CYP3A1 by GA. In conclusion, our findings demonstrated that both EX and GA exhibited significant hepato-protective effects against RTS-induced hepatotoxicity, mainly through the competitive inhibition of CYP-mediated metabolic activation of RTS.

## Introduction

**FIGURE 1:**
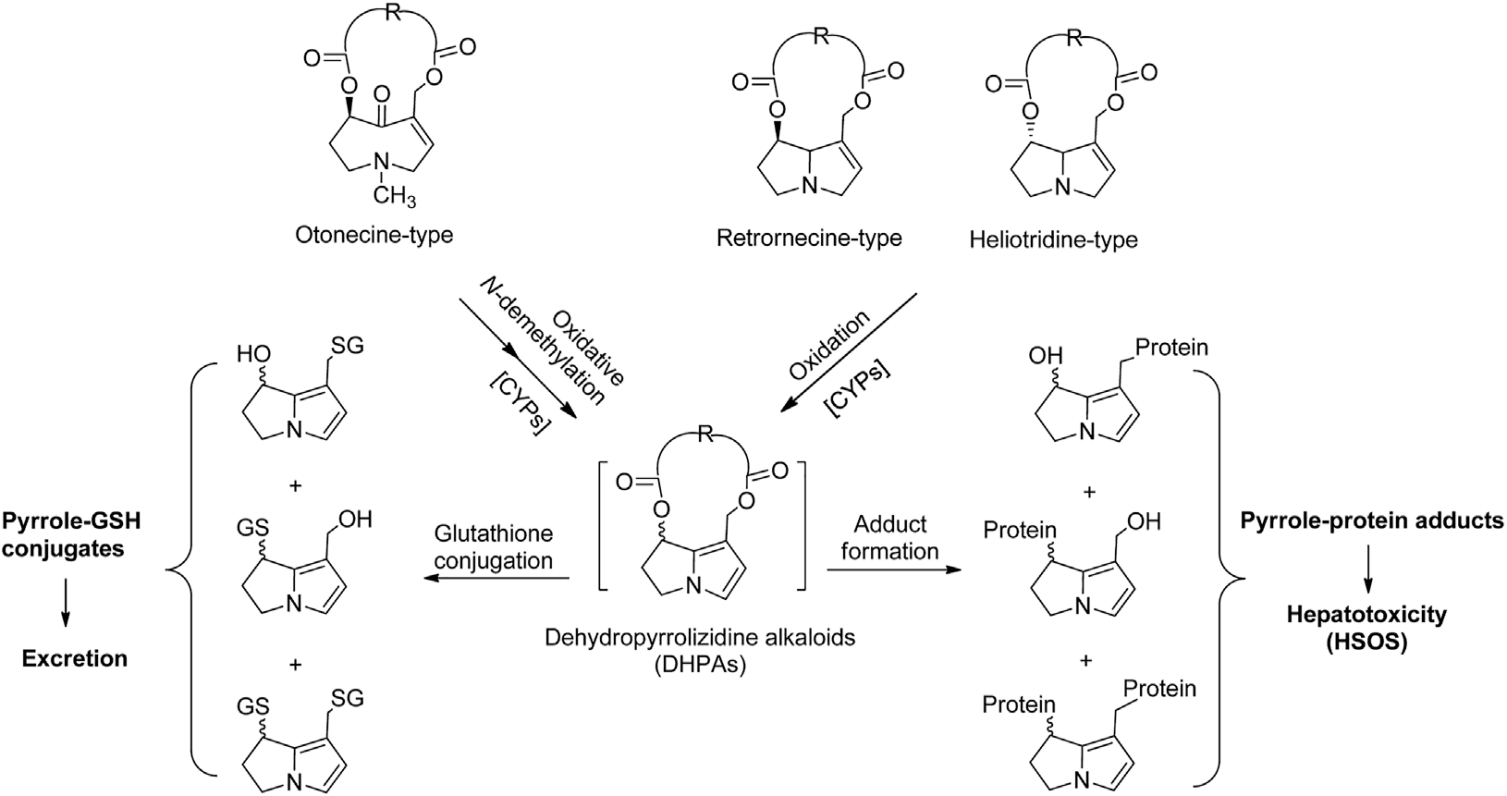
The scheme of hepatic metabolic activation of retronecine-, heliotridine-, and otonecine-type PAs to form dehydropyrrolizidine alkaloids (DHPAs), which either bind with glutathione as a detoxification pathway, or interact with proteins to generate pyrrole-protein adducts, leading to liver damage.

Pyrrolizidine alkaloids (PAs) are one of the most significant groups of phytotoxins widely presented in various plant species (He et al., 2021a). More than 660 PAs and their *N*-oxides have been identified in over 6,000 plants, which account for 3% of flowering plants (Dusemund et al., 2018; Schrenk et al., 2020). In addition, more than half of the identified PAs and their *N*-oxides have been reported to be hepatotoxic, carcinogenic, pneumotoxic, neurotoxic, and embryotoxic (Fu, 2017). PAs require metabolic activation in the liver to exert toxicity. Mediated by hepatic cytochrome P450 (CYPs), three toxic types (retronecine-type, heliotridine-type, and otonecine-type) of PAs generate reactive metabolites, dehydropyrrolizidine alkaloids (DHPAs), which form adducts with proteins (pyrrole-protein adducts) and cause dysfunction of critical proteins, thus damaging hepatic sinusoidal endothelial cells and leading to hepatotoxicity (Figure 1) (Ruan et al., 2014; Yang et al., 2016; Geburek et al., 2020; He et al., 2021b; Geburek et al., 2021). Among all CYP subfamilies, CYP3A and CYP2B isoforms have been identified as the major subfamily responsible for the metabolic activation of toxic PAs (Ruan et al., 2014). Alteration of activity of CYP3A subfamily, especially CYP3A4 isoenzyme, was reported to significantly affect the outcome of PA intoxication. For instance, phenobarbital, a CYP3A isozymes inducer, enhanced metabolic activation of riddelliine (a retronecine-type PA) and then increased the riddelliine‐induced liver toxicity (Kasahara et al., 1997). In addition, the human CYP3A4-overexpressed Madin Darby Canine Kidney (MDCK) cells were more susceptible to monocrotaline (a retronecine-type PA) compared to mock MDCK cells, due to the increased metabolic activation of monocrotaline (Tu et al., 2014).

PA-poisoning cases have been constantly reported across the world (Willmot and Robertson, 1920; Tandon et al., 1976; Zhuge et al., 2019; Ma et al., 2021; Zhu et al., 2021). Humans are exposed to PAs through misuse of PA-containing herbs (Dai et al., 2007; Yang et al., 2017) or PA-contaminated foodstuffs such as grains, honey, milk, and eggs (Mohabbat et al., 1976; Dussourd et al., 1989; Kakar et al., 2010; Bodi et al., 2014). Intake of high amounts of toxic PAs injured the protein membranes of hepatocytes, particularly liver sinusoidal endothelia cell (SECs), leading to life-threatening hepatic sinusoidal obstruction syndrome (HSOS), which presented with clinical manifestations such as tender hepatomegaly, hyperbilirubinemia, and ascites (Deleve et al., 2002; Gao et al., 2012; Teschke et al., 2021). This injury is confirmatively diagnosed by blood pyrrole-protein adducts, which are specific diagnostic biomarkers, supporting the causality evaluation using the updated Roussel Uclaf Causality Assessment Method (RUCAM) (Danan and Teschke, 2016; Teschke and Danan, 2020). Furthermore, our recent study discovered an unexpectedly extensive implication of PA exposure in patients with liver cancer, providing a clinical indication of PA-associated liver cancer (He et al., 2021c).

Liquorice (Glycyrrhizae radix et rhizome), the roots of *Glycyrrhiza uralensis* Fisch., *Glycyrrhiza glabra* L. or *Glycyrrhiza inflate* Bat., has long been used as a tonic herbal medicine in traditional Chinese medicine (TCM) due to its ability to harmonize unpleasant characteristics of other herbs or reduce the toxicities of certain herbs when used together in the herbal formulas (Wang et al., 2013; Wahab et al., 2021). In China, although there are no reports on the scientific rationale, liquorice is presented with PA-containing herbs in TCM compound formulas, yet no PA-poisoning cases have been reported with this combinational use. For example, Nin Jiom Pei Pa Koa, a very popular herbal formula used in Chinese communities worldwide for relief of minor discomfort in sore mouth/throat, contains both coltsfoot (a PA-containing herb) and liquorice. In addition, in Pharmacopeia of P.R. China (ChP 2020 Edition) six PA-containing herbs are listed and used in the preparation of many Chinese proprietary herbal products, and among them, 16 out of 35 are found to contain liquorice (Table 1). Furthermore, in the medical encyclopedia database, 68 compound TCM formulas and 12 TCM proprietary products were found to contain PA-containing herbs, and ∼53% of compounded TCM formulas and ∼25% of the TCM proprietary products contained liquorice, respectively (Table 1), suggesting the potential role of liquorice in detoxifying/harmonizing PA intoxication.

**TABLE 1:** Percentage of liquorice in PA-containing TCM proprietary products and Compound TCM formulas.

PA-containing botanical drug	TCM proprietary products in ChP 2020			TCM proprietary products in medical encyclopedia database			Compound TCM formulas in medical encyclopedia database		
	Liquorice		Percentage^a^	Liquorice		Percentage^a^	Liquorice		Percentage^a^
	−	+		−	+		−	+	
Senecionis scandentis hebra	4	0	0	7	1	14.3%	11	3	27.3%
Farfarae flos	14	12	85.7%	4	2	50.0%	19	14	73.7%
Eupatorii herba	2	0	0	1	0	0	16	3	18.8%
Arnebiae radix	8	3	37.5%	/	/	/	22	16	72.7%
Arnebiae radix^b^	7	1	14.3%	/	/	/	/	/	/
Total	35	16	45.7%	12	3	25.0%	68	36	52.9%

a The percentage of liquorice present in TCM, proprietary products/Compound TCM, formulas having PA-containing herbs.

b The herb is for external use.

Liquorice extract contains sugar, starch, resins, low levels of protein, and individual amino acids. Triterpene saponins and flavonoids are the main constituents isolated from *Glycyrrhiza* species. Glycyrrhizin (GL) is the predominant constituent of liquorice and biotransformed to its aglycone 18β-glycyrrhetinic acid (GA) in the body, and thus, GA has been confirmed as the primary bioactive form *in vivo* (Öztürk et al., 2017). Both GL and GA were reported to protect the livers from damages caused by carbon tetrachloride (Lee et al., 2007) and acetaminophen (Yan et al., 2016). Therefore, it is highly possible that liquorice may antagonize the toxic effects of PAs and relieve any potential liver damage caused by PAs. In the present study, we investigated the hepato-protective effect of liquorice extract (EX) and its primary bioactive ingredient GA on liver damage caused by retrorsine (RTS, a representative toxic PA) and delineated the underlying mechanism, in order to provide a scientific basis for the use of liquorice and/or GA as the antidote(s) for the prevention/detoxification of PA-induced liver injury.

## Materials and Methods

### Chemical and Reagents

RTS and GA (G10105, 97%) were purchased from Sigma-Aldrich Chemical Co. (St. Louis, MO, United States). Cocktail substrate and metabolite standards, NADPH tetrasodium salt, glucose-6-phosphate dehydrogenase (G-6-PD), glutathione (GSH), and all other chemicals, unless indicated, were purchased from Sigma-Aldrich Chemical Co. (St. Louis, MO, United States). Pyrrole-GSH conjugate, 7,9-diGSH-(±)-6,7-dihydro-7-hydroxy-1-hydroxymethyl-5*H*-pyrrolizine (7,9-diGS-DHP), was prepared as previously reported (Lin et al., 2000; Ma et al., 2015). HPLC-grade ethanol, acetone, formic acid, and acetonitrile were purchased from Merck (Darmstadt, Germany). Anti-CYP3A1 antibody was purchased from Santa Cruz Biotechnology (sc-53246, Santa Cruz, CA, United States). Horseradish peroxidase-conjugated goat anti-rabbit and goat anti-mouse were purchased from Cell Signaling Technology Inc. (Danvers, MA, United States). Anti-RECA-1 antibody (ab197727) and Alexa Fluor 488-conjugated goat anti-mouse IgG (ab150117) were supplied by Abcam (Cambridge, MA, United States).

### Preparation of Liquorice Aqueous Extract

Liquorice aqueous extract prepared from the root of *Glycyrrhizae glabra* L. (No. LC151018) was collected from Kazakhstan in Oct. 2015 and dried at 55°C for 12 h, and then authenticated by the co-author Prof. Yang YE’s group. The dried root of liquorice was extracted three times by refluxing with water (1:5 w/v) for 2 h per time. The water extract was then combined and lyophilized to provide liquorice extract (EX). EX was accurately weighed, completely dissolved in distilled water, and analyzed by HPLC-UV. The HPLC-UV analysis was performed on an Agilent 1100 liquid chromatograph system (Agilent Technologies, Inc., Palo Alto, United States). The chromatographic separation was performed on an Agilent C18 (4.6 × 250 mm, Shodex, Tokyo, Japan) column. The mobile phase was a mixture of water containing 1% formic acid (A) and acetonitrile (B), with a gradient elution as follows: 0–15 min, 75% A; 15–25 min, 25% A; 25–35 min, 75% A. The flow rate was set at 1 ml/min. The detection wavelength was 254 nm, and the injection volume was 20 µL. A representative HPLC-UV chromatogram of EX is shown in Supplementary Figure S1. The obtained EX was stored at -20°C before use.

### Animals and Treatments

Male Sprague Dawley rats (about 200–220 g) were supplied by the Laboratory Animal Service Centre at the Chinese University of Hong Kong. The animal room was maintained at 25 ± 1°C with a 12-h light-dark cycle and 55 ± 5% humidity. The rats had free access to standard rodent chow and water. All procedures were approved by the Animal Experimental Ethics Committee, the Chinese University of Hong Kong under the regulations of Hong Kong SAR government.

Rats were randomly divided into the following twelve groups: 1 and 2) Single- or Multiple-dose of distilled water groups (Vehicle), 3 and 4) Single-dose of RTS groups (RTS), 5 and 6) Single- or Multiple-dose of EX groups (SD-EX and MD-EX), 7 and 8) Single- or Multiple-dose of GA groups (SD-GA and MD-GA), 9) Single-dose of EX + RTS group (RTS-SD-EX), 10) Multiple-dose of EX + RTS group (RTS-MD-EX), 11) Single-dose of GA + RTS group (RTS-SD-GA), and 12) Multiple-dose of GA + RTS group (RTS-MD-GA). EX and GA were dissolved in distilled water at 37°C for oral administration with the dosage of EX and GA as 500 mg/kg and 50 mg/kg. RTS was dissolved in distilled water at room temperature to make the final concentration of 40 mg/kg. RTS was given following the last dose of distilled water/GA/EX. Detailed dosage regimens are described in Supplementary Figure S2. All rats were sacrificed at 48 h after the last dosing. Serum and liver samples were collected and stored at −80°C until use. The dose of RTS chosen for this study was based on our previous findings in that 40 mg/kg of RTS caused significant acute liver injury with moderate to severe SECs damage in male SD rats (Chen et al., 2021). According to the intake of the Compound Glycyrrhizin Tablets (containing 25 mg GL/per tablet), the suggested daily intake of GL is 225 mg/day for humans, which is approximately 20 mg/kg/day GA for rats. In our experiments, we used 50 mg/kg/day GA and 500 mg/kg/day EX (suggested daily intake of 5–20 g of liquorice root) for 5 consecutive days, which is close to the normal dose and has also been reported in many publications (Lin et al., 1999; Zhang et al., 2019; Yan et al., 2021).

For the toxicokinetic study, jugular vein cannulation was performed on rats on the day before drug administration. The right jugular vein was cannulated with a polyethylene tube (0.4 mm i.d. × 0.8 mm o.d., SIMS Portex, United Kingdom) for blood sampling. The rats were maintained in individual metabolic cages and fasted overnight with free access to water. Rats were orally dosed with GA (50 mg/kg) or distilled water (<5 ml/kg) 2 hours prior to RTS (30 mg/kg). After RTS dosing, blood samples (about 0.2 ml/sample) were collected at 5, 10, 20, 40, 60, 120, 240, 360, 480, 720, and 1440 min and placed in heparinized tubes. Saline (0.2 ml) containing 25 units of heparin/mL was injected after each blood sampling for compensation of blood withdrawal. The blood samples were then centrifuged at 3,000 x g for 30 min, and plasma samples were harvested and stored at −20°C until use.

### Histological and Biochemical Analysis

The largest lobe of the liver was sliced, fixed in 10% buffered-neutral formalin for 24 h, and embedded in wax. Sections of 5 µm in thickness were subjected to hematoxylin and eosin staining before being examined. Liver injury (necrosis, endothelial cell damage, and hemorrhage) was evaluated with a LEICA DM5000B Microscope (Leica, Heidelberg, Germany).

The serum alanine transaminase (ALT) level was measured by a kit obtained from Sigma-Aldrich Chemical Co. (St. Louis, MO, United States). The total bilirubin level was measured by the QuantiChromTM Bilirubin Assay kit according to the manufacturer’s instructions (BioAssay Systems, Hayward, CA, United States). Hepatic malondialdehyde (MDA) level was measured using a kit obtained from Nanjing Jiancheng Bioengineering Institute (Nanjing, China). Total GSH content in the liver was measured following a standard spectrophotometric method using Ellaman’s reagent.

### Immunofluorescence of Rat Endothelial Cell Antigen-1

The largest lobe of the rat liver was sliced, cold-embedded in Tissue-Tek OCT compound, and processed for immunohistochemistry according to the Abcam IHC frozen sections staining protocol. The tissue slices were incubated with rat endothelial cell antigen (RECA)-1 antibody and Alexa Fluor 488-conjugated goat anti-mouse IgG, and then stained with DAPI and observed under Olympus FluoViewTM FV1000 confocal microscope.

### Quantification of Pyrrole-Protein Adducts, 7,9-diGS-DHP, and RTS

The pyrrole-protein adducts were measured according to our previously developed method (Lin et al., 2011; Ruan et al., 2014). Briefly, 50 mg liver or 100 µL serum/plasma was mixed with acetone and centrifuged at 900 x g for 10 min. The precipitated protein was treated with silver nitrate (20 mg/ml) in ethanol containing 5% trifluoroacetic acid to release pyrrole moiety from the pyrrole-protein adducts. After centrifugation, an aliquot of the supernatant was incubated with 4-dimethylaminobenzaldehyde (20 mg/ml) at 56°C for 10 min. The mixture was filtered and subjected to LC-MS/MS analysis. 7,9-diGS-DHP was used as a standard with a concentration ranging from 0.2 to 2000 nM and underwent the same preparation process to construct a calibration curve. To measure the concentration of RTS and 7,9-diGS-DHP, liver, serum, plasma samples, or the incubation mixture were mixed with five volumes of acetone (for liver samples) or three volumes of acetonitrile (for serum and plasma samples) to precipitate the proteins. After centrifuging at 20,000 x g for 30 min, the supernatants were collected and analyzed by LC-MS/MS according to our previously developed methods (Ma et al., 2019; He et al., 2021a; Ma et al., 2021).

### Effects of GA on the Formation of 7,9-diGS-DHP and Different CYP Activities

To determine the effect of GA on the formation of pyrrole-GSH conjugate (7,9-diGS-DHP), GA (0, 2.5, 5, 10, 30, 50, and 80 μM, prepared in DMSO) or ketoconazole (KCZ, 25 μM, prepared in DMSO) was incubated with 200 µM RTS in the incubation mixture containing 1 mg/ml rat liver microsomes, 1 mM NADP^+^, 10 mM G-6-P, 5 mM MgCl_2_, 2 mM GSH, 1 U/ml G-6-PD and potassium phosphate buffer (0.1 M, pH 7.4). The reaction was initiated by adding RTS and stopped with the addition of ice-cold acetonitrile after 120 min incubation at 37°C. The mixtures were centrifuged, and supernatants were analyzed by LC-MS/MS.

A reported cocktail assay (Otten et al., 2011) was used for evaluating the effect of GA on different CYP activities by LC-MS/MS with high sensitivity and selectivity. The concentrations of probe substrates were listed in Supplementary Table S1. Various concentrations of GA (0, 2.5, 5, 10, 30, 50, and 80 µM) were added to the reaction mixture containing mixed CYPs probe substrates, NAPDH-regenerating system (5 mM MgCl_2_, 1 mM NADP^+^, 1 mM G-6-PD), and 1 mg/ml rat liver microsomes.

### Enzymatic Kinetic Study

The kinetic study was performed to determine the inhibitory mechanism of GA on rat CYP3A1. Briefly, GA (0, 10, 25 and 50 µM) was incubated in 200 µL incubation system containing 1 mg/ml rat liver microsomes, 5 mM MgCl_2_, 1 mM NADP^+^, 1 mM G-6-PD, and nifedipine (0.1, 0.5, 1, 2, 5, 10, 20, 50, 100, and 200 µM). The mixture was pre-incubated at 37°C for 15 min. The reaction was initiated by adding nifedipine for 45 min incubation at 37°C, and then quenched with ice-cold acetonitrile and centrifuged at 15,000 x g for 20 min. The supernatant was filtered through a 0.22 µm membrane filter before the LC-MS/MS analysis.

### LC-MS/MS Analysis

The LC-MS/MS analysis was performed on an Agilent 6460 Triple Quadrupole LC/MS System using a Waters Acquity BEH C18 column (2.1 × 100 mm, 1.7 mm). For the detection of pyrrole-protein adducts, the mobile phase of water containing 0.1% formic acid (A) and acetonitrile containing 0.1% formic acid (B) was used with a gradient elution as follows: 0–5 min, 35–95% B; 5.5–6 min, 95–35% B. The flow rate was 0.3 ml/min. The injection volume was 3 μL. For the detection of pyrrole-GSH conjugates and RTS, a gradient elution was used as follows: 0–1 min, 2% B; 1–7 min, 2–25% B, 7–7.5 min, 25–95% B. 7.5–10 min, 95% B. The flow rate was 0.3 ml/min. The injection volume was 5 µL. The mass spectrometer was operated in multiple reactions monitoring for data acquisition. The multiple reaction monitoring (MRM) transitions, fragmentor, and collision energy for 7,9-diGS- RTS, RTS, and pyrrole-protein adducts are listed in Supplementary Table S1.

For the cocktail assay, a gradient elution was used as follows: 20% A (0–1 min), 20–95% A (1–4 min), 95% A (4–6 min). The MRM transitions and linearity information for each substrate are indicated in Supplementary Table S2. The calibration curves of individual CYP substrates and their corresponding metabolites were constructed by plotting the peak area versus the spiked concentration.

### Immunoblot Analysis

Proteins were separated by gel electrophoresis and electrophoretically transferred to nitrocellulose paper. The nitrocellulose paper was then incubated with rat CYP3A1 antibody (1:1000) overnight and then reacted with horseradish peroxidase-conjugated secondary antibody. Bands were developed using an ECL chemiluminescence detection kit. Equal loading of proteins was verified by GAPDH. The semi-quantification of each band was measured by ImageJ software.

### Statistical Analysis

Data are expressed as mean ± SD. The Student’s *t* test was used for the comparison between two groups. The one-way ANOVA followed with a post hoc Bonferroni’s multiple comparison test was used for the comparison among multiple groups. Toxicokinetic parameters were calculated by noncompartmental methods using WinNonlin version 4.0 (Pharsight, Mountain View, CA, United States). The enzyme kinetic parameters were estimated from the best fit line using least-squares linear regression of the inverse substrate concentration versus the inverse velocity (Lineweaver-Burk plots), and the mean values were used to calculate the K_m_ and V_max_. The inhibition constant (*K*_*i*_) value of GA was determined by the secondary Lineweaver-Burk plots. The statistical significance was set as ^*^ *p* < 0.05, ^**^ *p* < 0.01 or ^***^ *p* < 0.001.

## Results

### EX and GA Protected Rats From RTS-Induced Liver Injury

**FIGURE 2:**
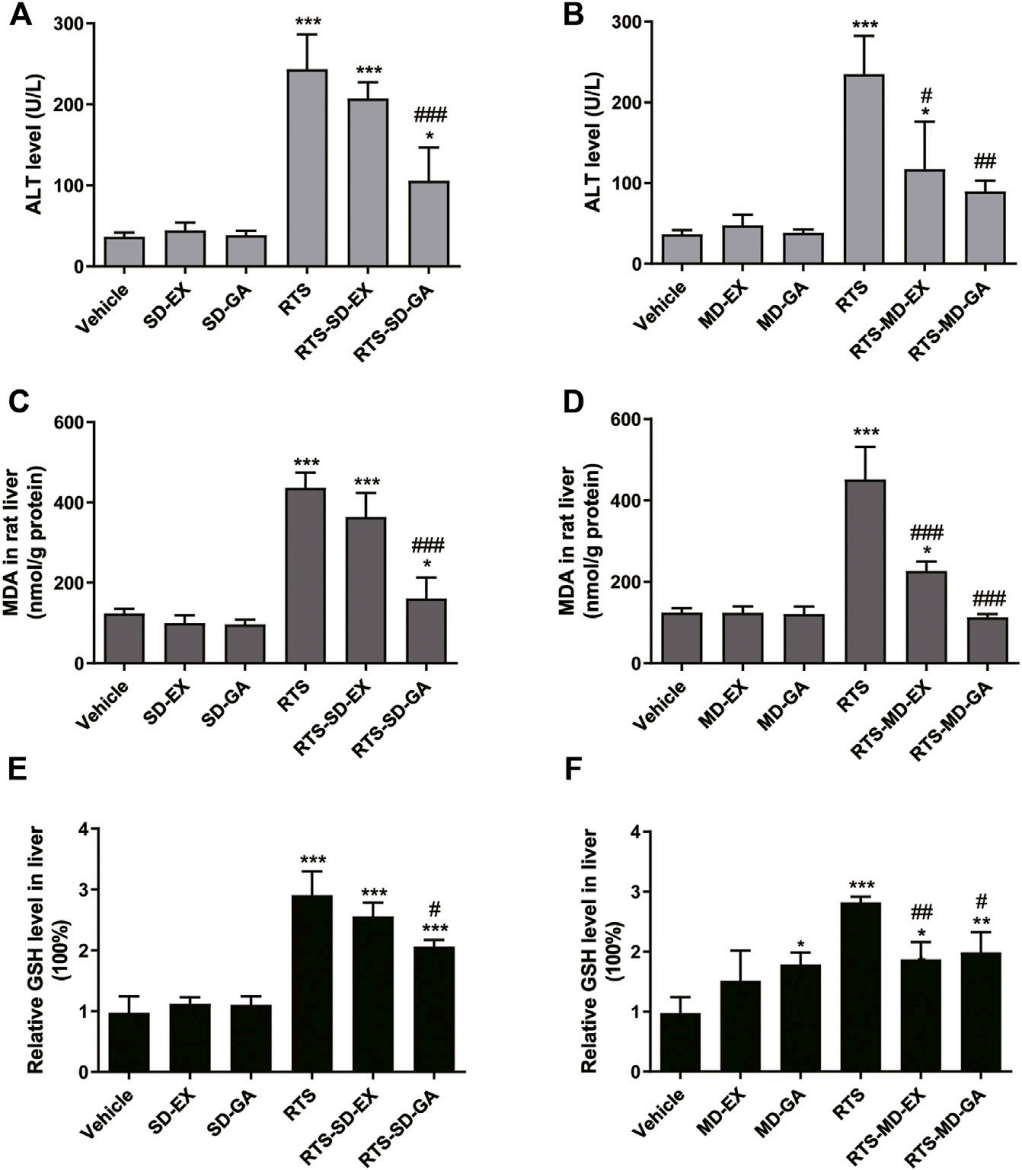
Single-dose (SD) and multiple-dose (MD) of EX or GA protected against RTS-induced liver injury. The activity/levels of serum ALT **(A,B)**, hepatic MDA **(C,D)**, and GSH **(E,F)** were measured in each group. ALT, alanine aminotransferase; MDA, malondialdehyde; GSH, glutathione. ^*^ *p* < 0.05, ^**^ *p* < 0.01, and ^***^ *p* < 0.001 versus vehicle group. ^#^ *p* < 0.05, ^##^ *p* < 0.01, and ^###^ *p* < 0.001 *vs*. the corresponding RTS group.

Compared with the vehicle groups, serum ALT and hepatic MDA levels were unchanged after both single and multiple administrations of EX and GA but elevated significantly at 48 h after RTS treatment. While a single dose of GA significantly reduced RTS-induced ALT and MDA elevations (Figures 2A,C), the multiple doses of both EX and GA also significantly attenuated these RTS-induced elevations (Figures 2B,D). In addition, hepatic GSH level increased significantly (2.82-fold, *p* < 0.001) at 48 h after RTS dosing as a feedback response to the activation of the antioxidant system, while this elevation was attenuated by the treatment of both EX and GA, especially their multiple dosage regimens (Figures 2E,F).

**FIGURE 3:**
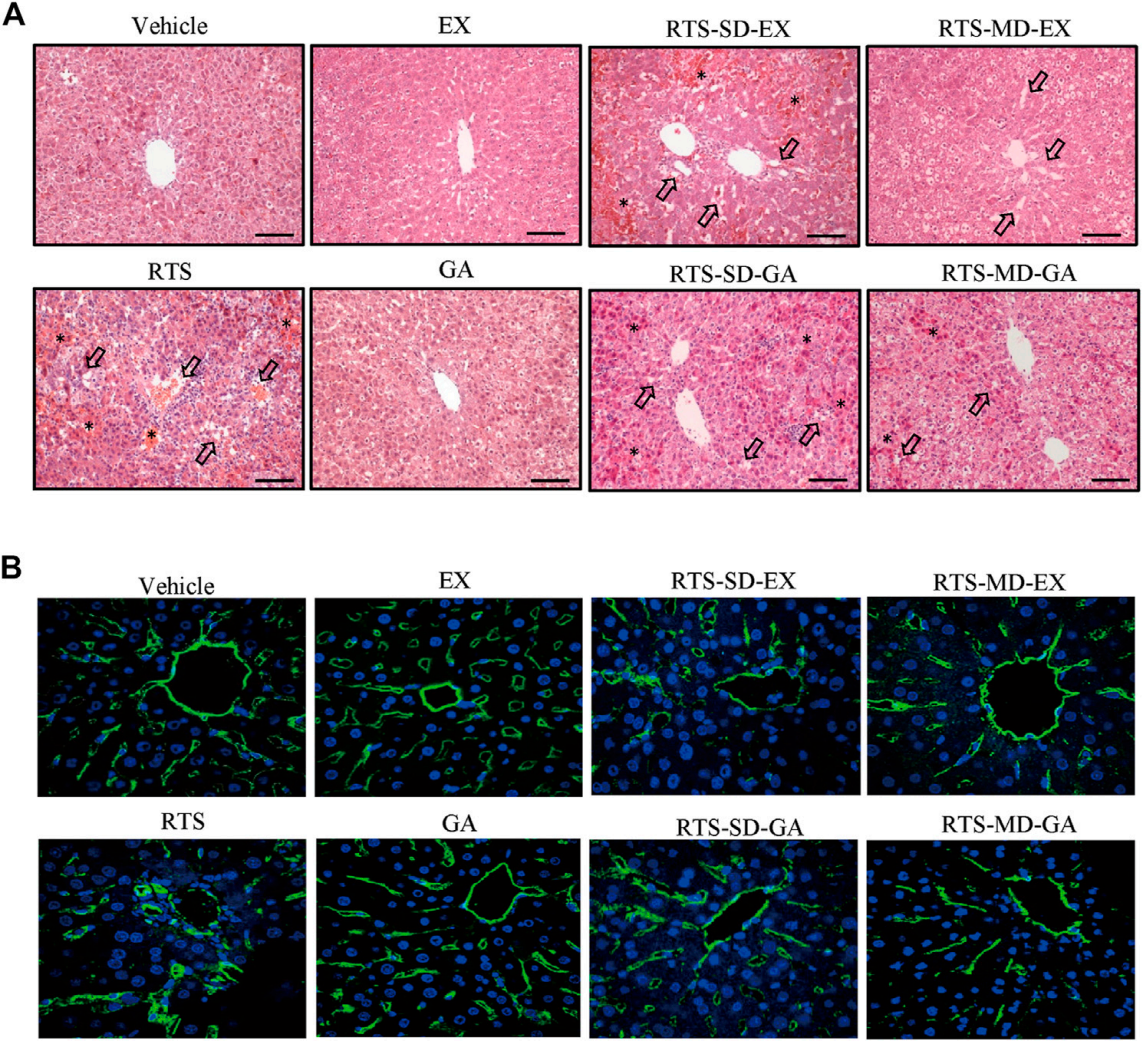
Single-dose and multiple-dose of EX or GA protected against RTS-induced liver damage. **(A)** Hematoxylin and eosin staining of liver. Arrows indicate the necrosis of hepatocytes. *, hemorrhage; black scale bar, 100 µm. **(B)** Immunostaining of RECA-1 in liver sections. Sinusoidal endothelial cells (SECs) in central vein and pericentral area were immunostained with anti-endothelial cell antibody (green), nucleus stained with DAPI (blue).RECA-1, rat endothelial cell antigen-1.

Protective effects of EX and GA on RTS-induced hepatotoxicity were also evaluated by histopathological examination. No obvious liver damage was observed in rats treated with EX or GA alone (Figures 3A,B). A single dose of RTS caused severe hepatotoxicity in rats, as demonstrated by massive hemorrhage and hepatocytes necrosis observed in the liver sections, whereas both single and multiple doses of EX or GA significantly ameliorated RTS-induced hemorrhage and necrosis (Figure 3A). The damage of sinusoidal endothelial cells was further measured by the immunofluorescence of RECA-1. Sinusoids, tightly distributed around central veins, were markedly damaged by RTS, and the injured sinusoids merged to form a large space with wide gaps among individual sinusoids, while this damage was significantly less in EX or GA treated groups (Figure 3B). The degrees of hemorrhage, sinusoidal dilation, and necrosis were assessed and scored (Table 2) according to a modified DeLeve’s system (Deleve et al., 1999) as summarized in Supplementary Table S3. All these biochemical and histopathological results clearly demonstrated that both EX and GA produced similar hepato-protective effects against PA-induced liver toxicity. Considering the individual hepato-protective effect of EX or GA multiple treatments exhibited a better protective effect than the single treatment scenario.

**TABLE 2:** Histological scoring of liver damage in the study of protective effect of EX and GA against RTS intoxication.

Group	Scoring parameters^a^								Overall^b^
	Central vein endothelial damage	Subendothelial hemorrhage	Sinusoidal hemorrhage	Sinusoidal dilation	Coagulative necrosis	Apoptotic bodies	Subendothelial fibrosis	Sinusoid fibrosis	
Vehicle	0	0	0	0	0	0	0	0	0
EX	0	0	0	0	0	0	0	0	0
GA	0	0	0	0	0	0	0	0	0
RTS	2-3	2-3	2	2-3	2	1	0	0	11-14
RTS-SD-EX	2-3	2	2	2	1-2	0	0	0	9-11
RTS-MD-EX	2	2	1	2	1	0	0	0	8
RTS-SD-GA	1	1-2	1	2	1	0	0	0	6-7
RTS-MD-GA	1	0-1	0-1	1-2	0	0	0	0	2-5

a Each parameter is graded on a four-point system: 0-absent, 1-mild, 2-moderate, and 3-severe.

b Overall represents the sum of the scores from all individual parameters.

### EX and GA Inhibited the Formation of Pyrrole-Protein Adducts

**FIGURE 4:**
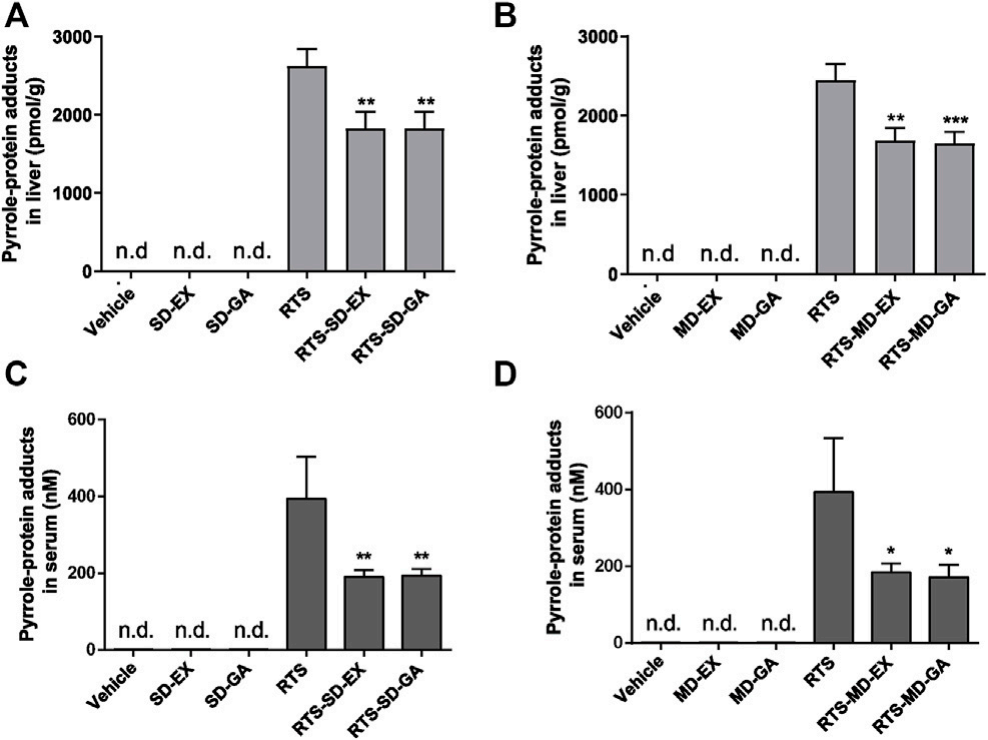
Single-dose and multiple-dose of EX or GA inhibited metabolic activation of RTS both *in vivo* and *in vitro*. Formation of pyrrole-protein adducts in the liver **(A,B)** and serum **(C,D)** in rats. Data are expressed as mean ± SD (*n* = 4). ^*^ *p* < 0.05, ^**^ *p* < 0.01, and ^***^ *p* < 0.001 versus the corresponding RTS group.

The formation of pyrrole-protein adducts is a critical initiating event of RTS-induced hepatotoxicity. As shown in Figure 4, the formation of pyrrole-protein adducts in the liver was significantly reduced by both single (Figure 4A) and multiple (Figure 4B) treatments with GA and EX to similar levels. Similarly, the serum pyrrole-protein adducts levels were also significantly reduced with the treatment of EX and GA in both single and multiple treatments to the same extent (Figures 4C,D). The following studies to delineate the mechanism underlying protective effect on RTS-induced hepatotoxicity were carried out with GA that is reported as the primary bioactive form *in vivo* (Öztürk et al., 2017).

**FIGURE 5:**
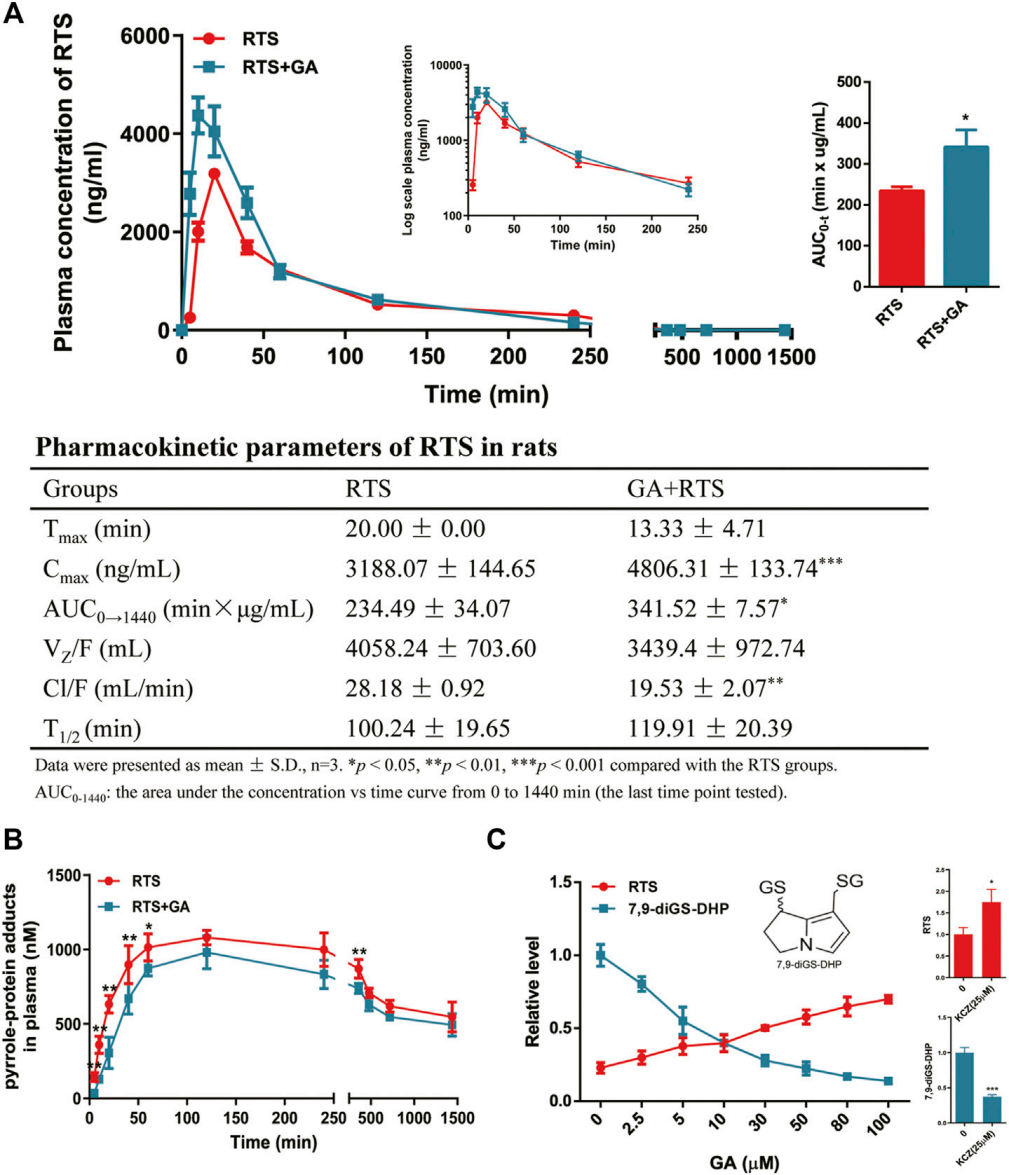
GA inhibited metabolic activation of RTS both *in vivo* and *in vitro*. Plasma kinetic profiles, AUC, and pharmacokinetic parameter of RTS **(A)** and plasma kinetic profiles of pyrrole-protein adducts **(B)** in RTS-treated rats orally dosed with vehicle or GA. Concentration changes of RTS and 7,9-diGS-DHP in the rat liver microsome incubation of RTS with GA (0–100 µM) or ketoconazole (KCZ, 25 µM) **(C)**. Data are expressed as mean ± SD (*n* = 3). ^*^ *p* < 0.05, ^**^ *p* < 0.01, and ^***^ *p* < 0.001 versus the corresponding RTS group.

For the study of the effect of GA on the toxicokinetic profile of RTS, rats treated with a single dose of GA and RTS exhibited significantly higher plasma *C*_max_ (4806.31 ± 133.74 ng/ml *vs*. 3188.07 ± 144.65 ng/ml, *p* < 0.001) and AUC_0–1440min_ (341.52 ± 7.57 min×µg/mL *vs*. 234.49 ± 34.07 min×µg/mL, *p* < 0.05) of RTS compared to the group treated with RTS alone (Figure 5A). In addition, the kinetic profile of pyrrole-protein adducts revealed an inhibitory effect of GA on the formation of pyrrole-protein adducts (Supplementary Table S4). In RTS + GA group, GA significantly reduced AUC_0–1440min_ of pyrrole-protein adducts to 856.49 ± 11.05 min × µM (16.2% reduction) compared to that (1022.49 ± 11.05 min × µM) in RTS group (Figure 5B). The results indicated that GA significantly inhibited the metabolic activation of RTS, leading to the reduced formation of pyrrole-protein adducts in rats.

Furthermore, the *in vitro* incubation with rat liver microsomes was also performed to confirm the inhibitory effect of GA on the metabolic activation of RTS. The dehydro-RTS, produced from metabolic activation of RTS by rat liver microsomes, was trapped by GSH to form pyrrole-GSH conjugate (7,9-diGS-DHP), since our previous study demonstrated that pyrrole mono-GSH adducts could not be detected in the blood of rats dosed with RTS (Lian W. and Lin G. 2017). The formation of 7,9-diGS-DHP after co-incubation with GA or ketoconazole (KCZ, CYP3A1 inhibitor) was then detected. GA significantly inhibited the formation of 7,9-diGS-DHP in a concentration-dependent manner with the estimated IC_50_ value of 5.07 µM, while in parallel, the concentration of intact RTS increased along with the concentration of GA (Figure 5C). In addition, the formation of 7,9-diGS-DHP was significantly reduced by 62% with the co-incubation of KCZ (25 µM) (Figure 5C). All the results confirmed that GA significantly inhibited metabolic activation of RTS, and thus reduced the production of toxic metabolite dehydro-RTS evidenced by the decreased formation of 7,9-diGS-DHP.

### GA Inhibited Activity Rather than Expression of CYPs

**FIGURE 6:**
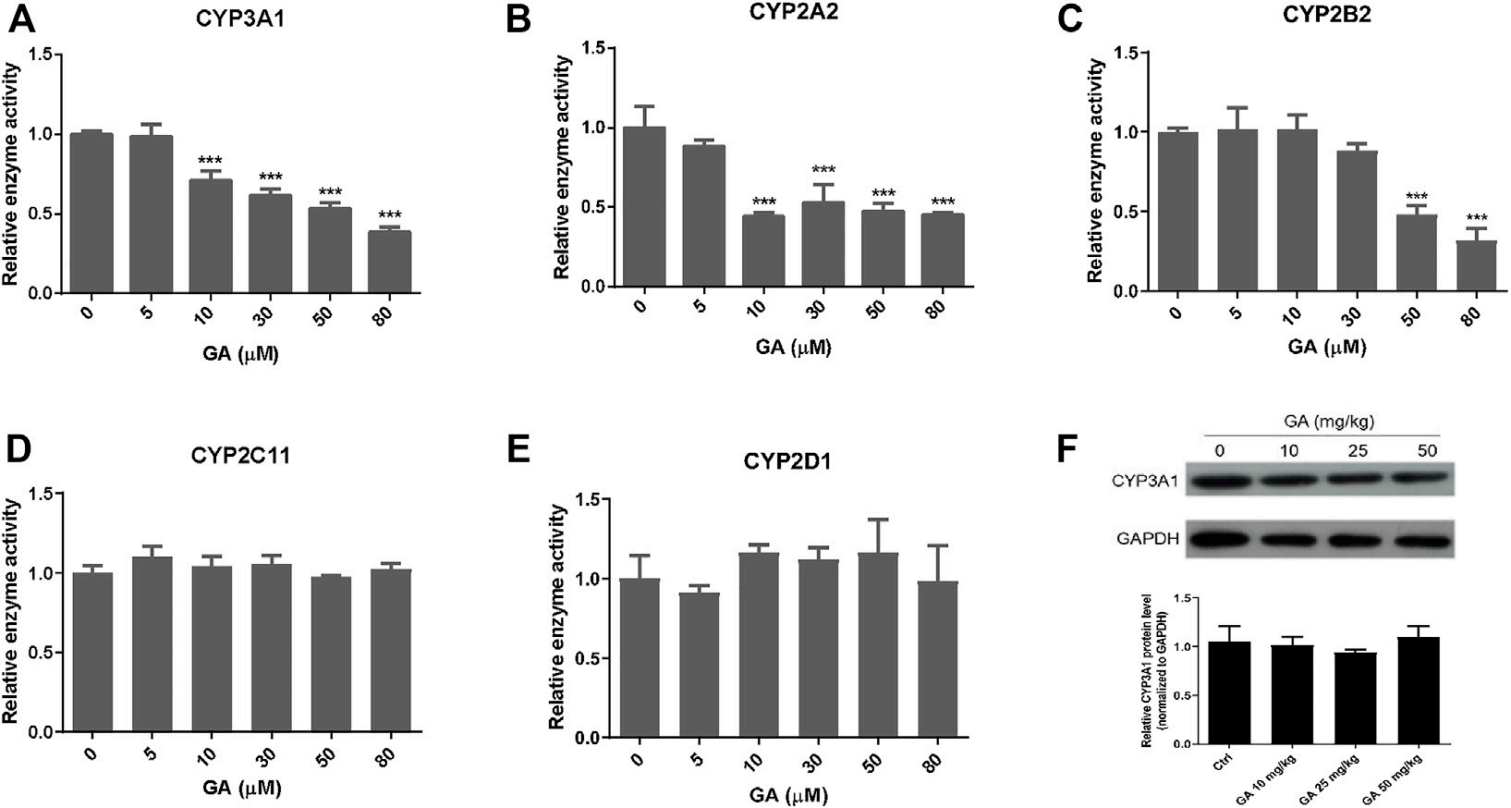
The effect of GA on the activity and expression of CYPs. **(A–E)** Inhibitory effects of GA on the activities of CYP3A1, CYP2A2, CYP2B2, CYP2C11, and CYP2D1 measured by cocktail assay *in vitro*. **(F)** Protein expression of CYP3A1 in the liver measured by western blot. Specific band intensity was quantified and normalized to GAPDH. Rats were orally administrated with different doses of GA (0, 10, 25, 50 mg/kg) for 5 consecutive days and sacrificed at 24 h after the last dose. Data are expressed as mean ± SD (*n* = 3). ^***^ *p* < 0.001 versus the corresponding GA (0 µM) group.

It is well-known that metabolic activation of PAs is mediated by hepatic CYPs, thus the effects of GA on the activity and protein expression of CYPs were determined by the cocktail assay. The results showed that GA inhibited the activities of rat CYPs, in particular CYP3A1, CYP2A2, and CYP2B2 (Figure 6). CYP3A1 (homologue to human CYP3A4), the predominant CYP isoform responsible for the metabolic activation of RTS, was inhibited by GA in a concentration-dependent manner with 68.5% inhibition at the highest concentration (80 µM) of GA tested (Figure 6A). For other tested CYPs which play minor roles in the metabolism of RTS (Ruan et al., 2014), the activities of CYP2A2 and CYP2B2 were also reduced by GA (Figures 6B,C), while the activities of CYP2C11 and CYP2D1 were not altered by GA (Figures 6D,E). Furthermore, the effect of GA on protein expression of CYP3A1 was tested in rats, and the results revealed that multiple oral administration of GA once a day for 5 consecutive days did not affect the protein expression level of rat hepatic CYP3A1 (Figure 6F), further confirming that only the activity but not expression of CYP3A1 could be inhibited by GA.

### GA Competitively Inhibited Enzyme Activity of CYP3A1

**FIGURE 7:**
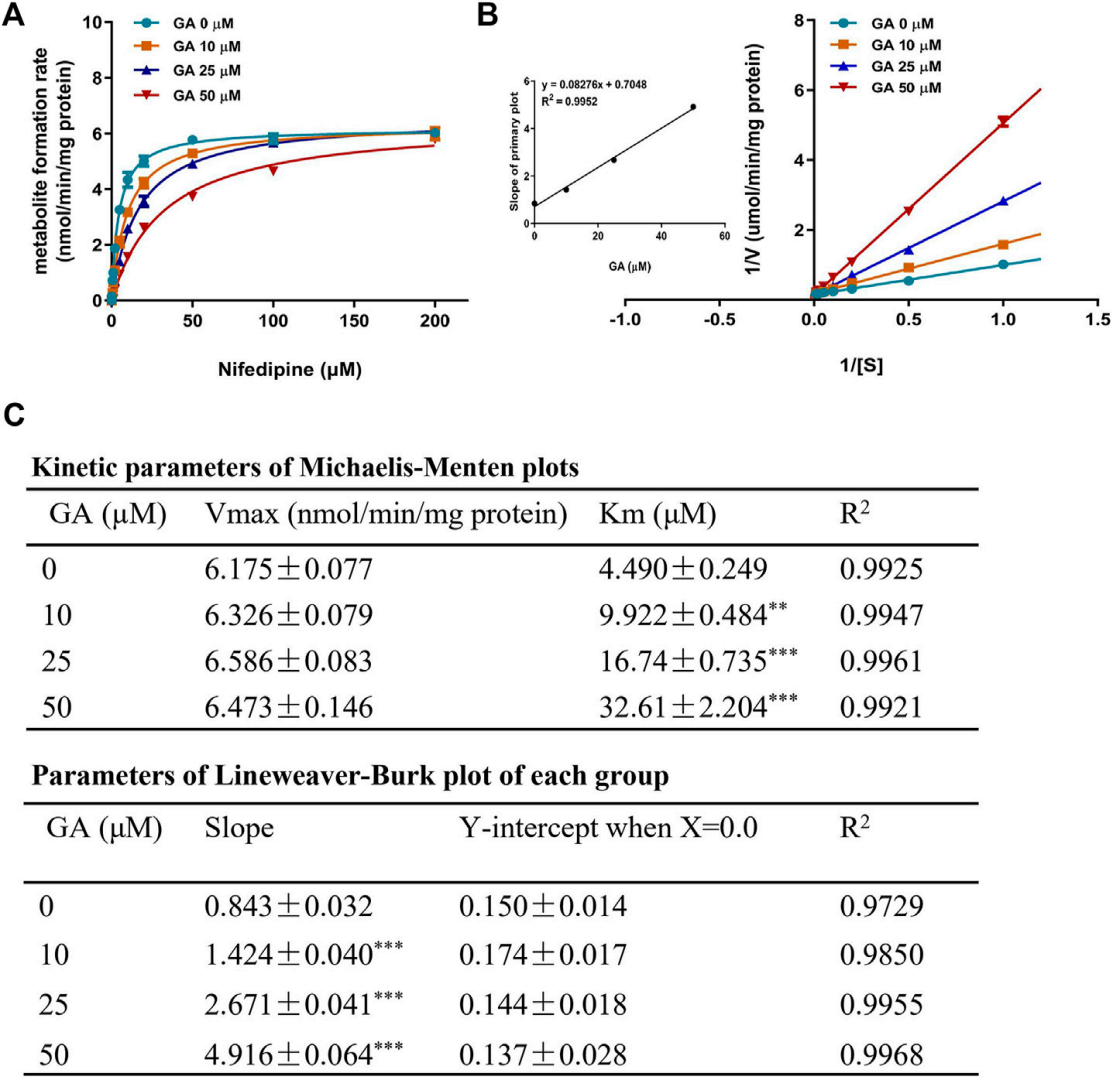
Michaelis-Menten kinetic plot **(A)** and Lineweaver-Burk plot **(B)** for *K*_*i*_ in the inhibition of rat CYP3A1-mediated metabolism of probe substrate (nifedipine, 0.1–200 µM) by GA (0, 10, 25, 50 µM) in rat liver microsomes. The kinetic parameters of Michaelis-Menten plot and Lineweaver-Burk plot **(C)**. Data are expressed as mean ± SD (*n* = 3). ^**^ *p* < 0.01 and ^***^ *p* < 0.001 versus the corresponding GA (0 µM) group.

To further explore the inhibitory mechanism of GA towards CYP3A1, an enzyme kinetic study was performed using nifedipine as the probe substrate for rat CYP3A1. The results from the Michaelis-Menten curve (Figure 7A) demonstrated that GA inhibited CYP3A1-mediated nifedipine metabolism with *K*_m_ value increased along with the increase in GA concentrations (Figure 7C). Furthermore, the Lineweaver-Burk plot (Figure 7B) indicated that GA concentration-dependently altered the slope but did not affect the *Y*-intercept, and the slope of each curve correlated well with the concentration of GA (*R* ^2^ = 0.9952) with *K*_i_ value of 8.52 µM (Figure 7B). All the results demonstrated that GA competitively inhibited the enzyme activity of CYP3A1.

## Discussion

Liquorice has been widely used in TCM for thousands of years to ameliorate liver damage associated with a number of clinical disorders. One of the most important known components of liquorice extract is GL, which has been explicitly recommended as a hepatoprotective drug by several guides (Wu et al., 2021; Wu et al., 2008). When GL is hydrolyzed, almost 100% of GL is irreversibly transformed into 18β-GA in the gastrointestinal tract by bacterial *ß*-*D*-glucuronidase after it is orally administrated (Takeda et al., 1996). Therefore, GA is the main metabolite of GL and the main active component of liquorice after oral administration. In the present study, we found that both single and multiple doses of EX or GA exerted protective effects against RTS-induced liver damage, as evidenced by ameliorated liver hemorrhage and hepatocytes necrosis, and lowered ALT levels (Figure 2 and Figure 3). Moreover, rats treated with EX or GA with both single and multiple treatments exhibited reduced oxidative stress with lower hepatic MDA levels when compared to those treated with RTS alone (Figures 2C,D), demonstrating the hepato-protective effect of both EX and GA against RTS-induced liver toxicity.

Pathogenetic studies of drug-induced liver injury (DILI) and herb-induced liver injury (HILI) have substantially increased within the past years. However, the clinical diagnosis of DILI/HILI remains a challenge. The updated RUCAM provides a robust quantitative diagnosis approach to assess the causality of suspected DILI and HILI cases (Danan and Teschke, 2016). Among the recent comprehensive review applied the RUCAM for causality assessment in 95885 cases of liver injury including 81856 DILI and 14029 HILI cases, 28 HSOS cases were demonstrated to be caused by PAs (Teschke and Danan, 2020). In China, the RUCAM is also recommended in the Chinese Society of Hepatology guidelines for the diagnosis and treatment of DILI. In addition, PA exposure biomarkers such as pyrrole-protein adducts, pyrrole-DNA adducts, and pyrrole-amino acid adducts have also been demonstrated to be specific and diagnostic biomarkers for the clinical diagnosis of suspected PA-induced HSOS cases (Ruan et al., 2015; Ma et al., 2019; He et al., 2021d; Ma et al., 2021). In our study, RTS caused typical HSOS with the disarray and necrosis of SECs around portal areas and central veins in rats (Figure 3B). Both single and multiple doses of EX or GA significantly attenuated the SEC damage, and multiple doses showed a stronger protective effect than the single dose (Figure 3B), In addition, both single- and multiple-dose of GA significantly inhibited the formation of pyrrole-protein adducts in the liver, providing firm evidence for the protective effect of EX and GA against RTS intoxication, especially reducing the risk of HSOS.

It has been reported that GL has poor oral bioavailability in both humans and rats, with very low levels after a single oral dose in the range of 100–1600 mg/kg (Gunnarsdóttir and Jóhannesson, 1997). However, GA is readily detected in plasma following the ingestion of GL or liquorice extract by rats and humans. In addition, the plasma peak of GA are lower and occur later when GL is administrated in liquorice extract than an equivalent dose of GL as a pure compound, suggesting a potential different hepatoprotective effect of EX and GL at the same dosage (Hou et al., 2005; Isbrucker and Burdock, 2006). Interestingly, results produced by EX or GA demonstrated very similar degrees of detoxification effect on RTS-induced hepatotoxicity with higher potencies via their multiple treatments than the single dosing (Figures 2–4). Apparently, these observations indicated that 1) almost all GL in EX was biotransformed to GA in the body, with the consideration of the doses of EX (500 mg/kg containing 10.82% of glycyrrhizin) and GA (50 mg/kg); 2) GA played a key role in detoxification; and 3) multiple treatments of GA exhibited a better hepato-protective effect than the single dose, suggesting a repeated/continuous treatment regimen of GA for better detoxification of PAs. Therefore, GA was then used in our following studies to delineate its underlying mechanism.

The cytochrome P450 (CYP) plays a vital role in the metabolism of toxic PAs. Dehydro-PAs, generated by CYP-mediated oxidation, are either detoxified by forming pyrrole-GSH conjugates followed by degradation and urinary/biliary excretion, or covalently bind to macromolecules, such as proteins, and cause massive hepatic necrosis. The clinical focus is on the most active CYP isoforms such as CYP3A4, CYP3A5, and CYP2A6 which show a striking difference in their substrate specificities, with the possible consequence that the degree of liver toxicity may be variable depending on the PA types and the specific CYP isoforms involved (Teschke et al., 2021). In general, most toxic PA types including retrorsine, riddelliine, senecionine, senkirkine, and lasiocarine are mainly metabolized by CYP3A4, which account for 30–50% of the hepatic CYP isoforms (Teschke et al., 2021). While PAs such as monocrotaline are mainly metabolized by the CYP2A6 (Suparmi et al., 2020). In the present study, because CYP3A4 is the predominant CYP isoform accounting for the RTS intoxication, we, therefore, focus on the study of the interaction between GA and CYP3A4 in our animal and cell models.

Previously, liquorice extract and its constituents have been reported to inhibit the activity of certain CYP isoforms (Kent et al., 2002; Tsukamoto et al., 2005; Pandit et al., 2011). For example, the methanol extract of liquorice was shown to inhibit CYP3A4 recombinant enzyme activity (Tsukamoto et al., 2005). A study using midazolam as a probe substrate revealed that GA decreased CYP3A4 activity in the human liver microsomes (Lv et al., 2015). In our toxicokinetic study, GA was found to significantly inhibit the metabolic activation of RTS and the formation of pyrrole-protein adducts (Figures 5A,B). Further *in vitro* cocktail assay revealed that GA significantly inhibited rat CYP3A1 (ortholog to human CYP3A4) activity in a concentration-dependent manner with 68.5% inhibition at the highest concentration (80 µM) tested (Figure 6). In addition, a competitive inhibitory mechanism of GA towards rat CYP3A1 was delineated by using standard Michaelis-Menten and Lineweaver-Burk plots (Figure 7). Therefore, our findings demonstrated that direct and competitive inhibition of rat CYP3A1 by GA was the predominant reason accounting for its protection against PA intoxication. On the other hand, with the observation of a better detoxification effect produced by the repeated doses of GA (Figure 6; Table 2), other mechanism contributed by the multiple treatments of GA is unknown and encouraged for further investigation.

Furthermore, it was found that *K*_m_ of nifedipine for rat CYP3A1 decreased along with the increasing concentration of GA (Figure 7C), revealing that GA and nifedipine competitively occupied the same active site of rat CYP3A1. We then used a computational docking model to investigate the molecular binding mode of GA with human CYP3A4 (Grosdidier et al., 2011). The results obtained from the SwissDock website showed that the energy of full fitness and estimated free energy of GA (-2468.20 kcal/mol, −6.7 kcal/mol) was comparable to those of KCZ (-2432.84 kcal/mol, −8.21 kcal/mol), a well-characterized CYP3A4 inhibitor causing a type II spectrum upon binding and indicating a direct heme binding and complex formation (Ekroos and Sjögren, 2006). The findings suggested that these two molecules might exhibit a similar distance towards the heme group of CYP3A4 and form potential interaction with this site. In addition, results obtained from AUTODOCK software further demonstrated that the keto group of GA located in the polar pocket of CYP3A4 through forming hydrogen bonds with the amino acid Arg-106 and Arg-372 on the side chain of CYP3A4 (Supplementary Figure S3). The data suggest that GA may interact with the CYP3A4 binding domain similar to that of KCZ, which interacts with the side chains of Arg-372, Arg-106, and Glu-374 by hydrophobic interactions such as *π*-stacking (Ekroos and Sjögren, 2006). Further studies are warranted to confirm the interaction pattern and binding domain of GA towards human CYP3A4.

Modern and traditional herbal medicines commonly used some PA-containing herbal products as medicinal plants to treat patients with less serious ailments, although the efficacy for most indications is insufficiently reported due to their traditional usage with the lack of randomized controlled trials in modern medical practice. Medical plants containing PAs are widely described in Europe, United States, Canada, China, etc. PA-producing medical pants belong to the plant families such as Boraginaceae, Asteraceae, Apiaceae, and Leguminosae, and their plants produce a high variability of PA profiles (Roeder, 2000). In the normal practice of TCM, these PA-containing herbs are commonly combined with other herbs in a single prescription in TCM. Liquorice, as a widely used herbal medicine applied in both medicinal and confectionery sectors, is regarded as a unique ‘guide drug’ to enhance the effectiveness of other ingredients, to reduce toxicity, or improve flavor in almost half of Chinese herbal formulas/prescriptions (Wang et al., 2013). Based on our study, we found that liquorice is present in almost half of the formulas which contain PA-containing herbs (Table 1), and no PA-poisoning cases have been reported under such combinational use, which may suggest the effect of liquorice against potential PA poisoning, and is warranted for future in-depth investigations.

In conclusion, the present study demonstrated that EX, especially its major bioactive ingredient GA, protected the liver from RTS, a representative toxic PA, induced toxicity. Furthermore, our results also delineated the underlying mechanism of such detoxification via competitive inhibition of GA on rat CYP3A1 (ortholog to human CYP3A4), resulting in the inhibition of CYP3A1/3A4-catalyzed metabolic activation of PAs followed by the toxic metabolites-mediated hepatotoxicity. All the findings provided a scientific rationale for the current practice of combining liquorice with PA-containing herbs, and also for the recommendation of future use of liquorice or GA for prevention of PA intoxication.

## Data Availability

The original contributions presented in the study are included in the article/Supplementary Material, further inquiries can be directed to the corresponding author.
