# Supplementary material for: Liquorice Extract and 18β-Glycyrrhetinic Acid Protect Against Experimental Pyrrolizidine Alkaloid-Induced Hepatotoxicity in Rats Through Inhibiting Cytochrome P450-Mediated Metabolic Activation
Source: Front Pharmacol. 2022 Mar 16;13:850859. doi: 10.3389/fphar.2022.850859 (PMC8966664; doi:10.3389/fphar.2022.850859)
Supplement: Supplementary file 1 [file DataSheet2.PDF]

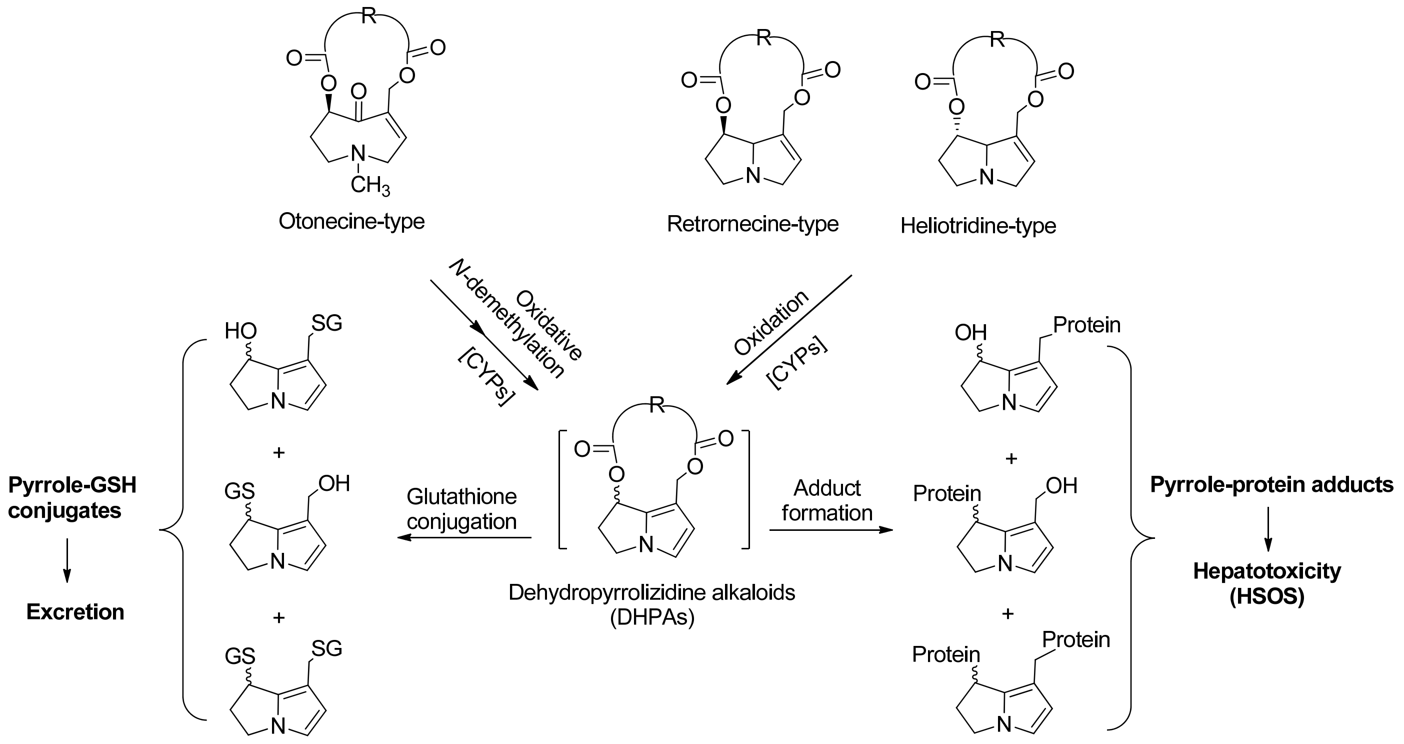

Otonecine-type
Retrornecine-type
Heliotridine-type
Oxidative
N-demethylation
[CYPs]
Oxidation
[CYPs]
Pyrrole-GSH conjugates
Excretion
Glutathione conjugation
Dehydropyrrolizidine alkaloids (DHPAs)
Adduct formation
Pyrrole-protein adducts
Hepatotoxicity (HSOS)

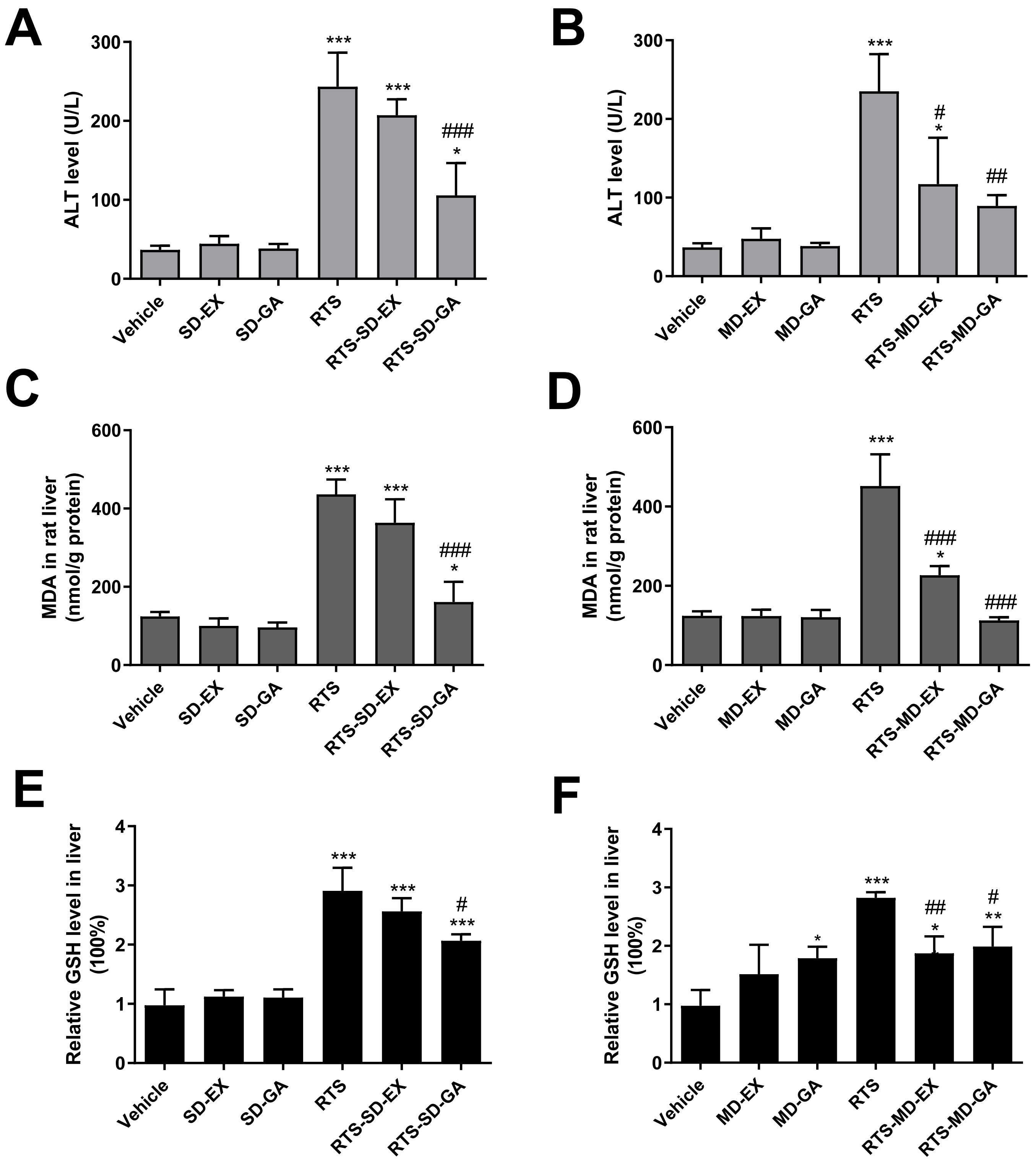
A
B
C
D
E
F
ALT level (U/L)
MDA in rat liver (nmol/g protein)
Relative GSH level in liver (100%)
Vehicle
SD-EX
SD-GA
RTS
RTS-SD-EX
RTS-SD-GA
MD-EX
MD-GA
RTS-MD-EX
RTS-MD-GA
***
*
#
##
###

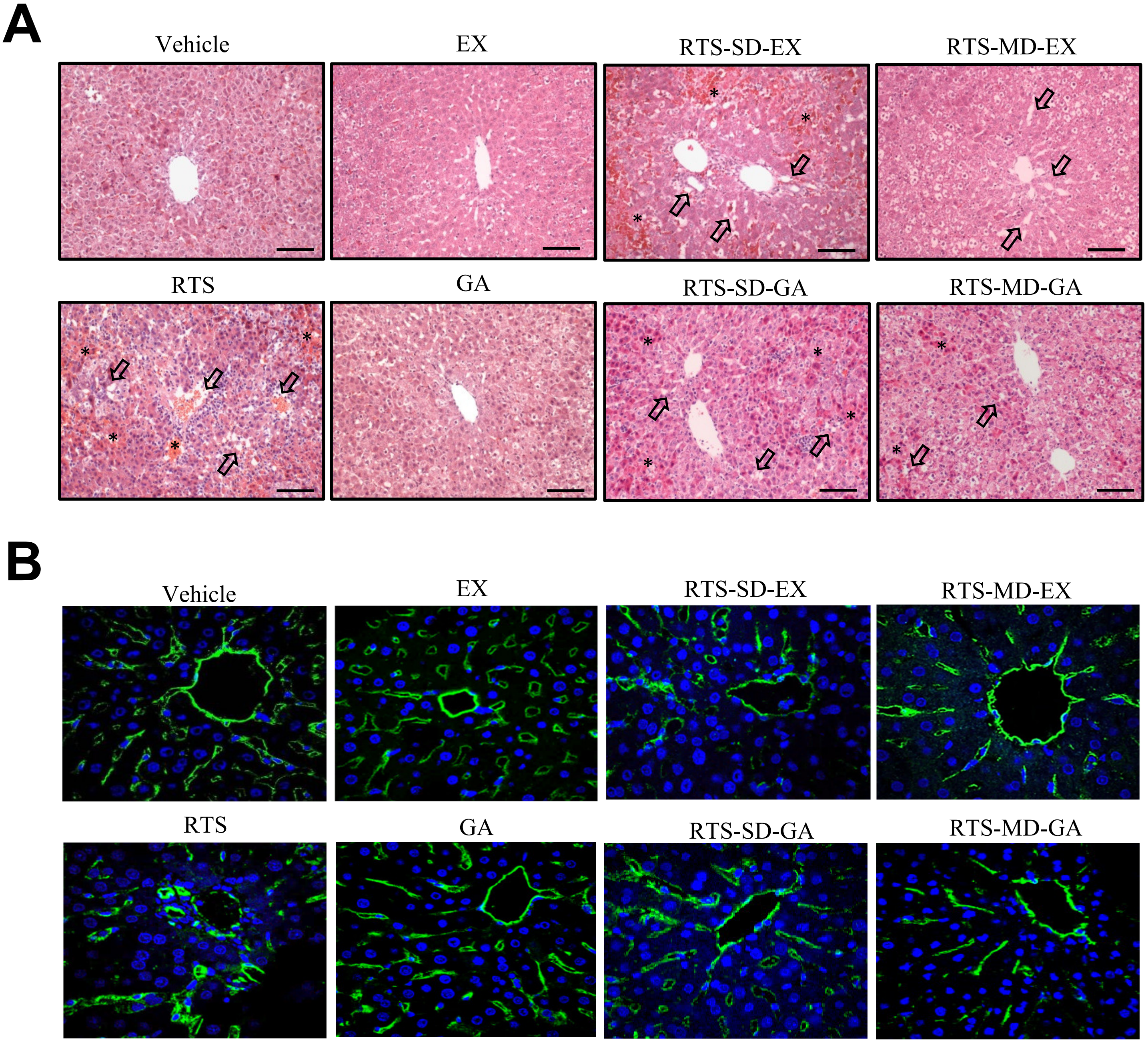

A
Vehicle
EX
RTS-SD-EX
RTS-MD-EX
RTS
GA
RTS-SD-GA
RTS-MD-GA
B
Vehicle
EX
RTS-SD-EX
RTS-MD-EX
RTS
GA
RTS-SD-GA
RTS-MD-GA

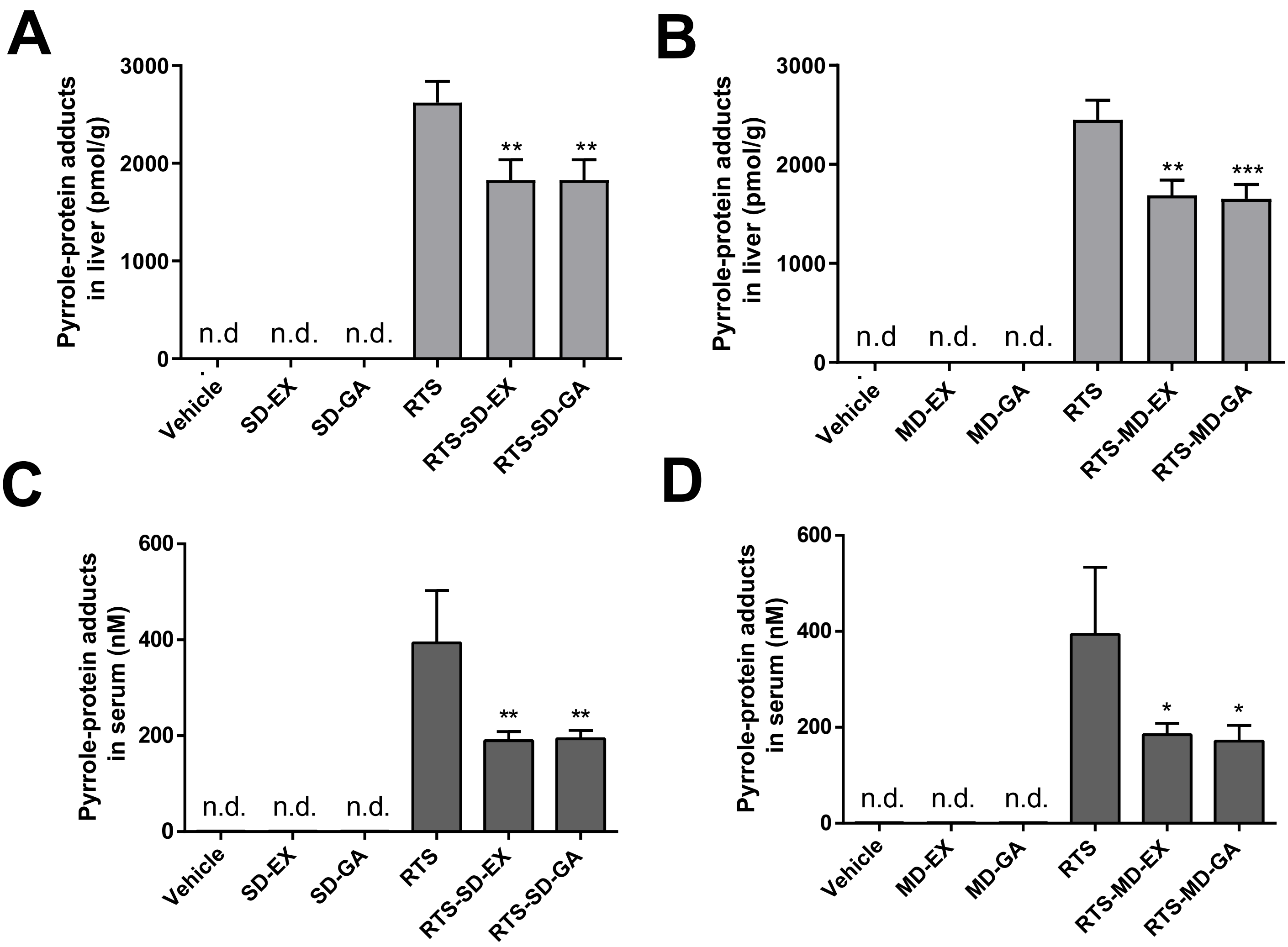
A
Pyrrole-protein adducts in liver (pmol/g)
3000
2000
1000
0
n.d
n.d.
n.d.
**
**
Vehicle
SD-EX
SD-GA
RTS
RTS-SD-EX
RTS-SD-GA
B
Pyrrole-protein adducts in liver (pmol/g)
3000
2000
1000
0
n.d
n.d.
n.d.
**
***
Vehicle
MD-EX
MD-GA
RTS
RTS-MD-EX
RTS-MD-GA
C
Pyrrole-protein adducts in serum (nM)
600
400
200
0
n.d.
n.d.
n.d.
**
**
Vehicle
SD-EX
SD-GA
RTS
RTS-SD-EX
RTS-SD-GA
D
Pyrrole-protein adducts in serum (nM)
600
400
200
0
n.d.
n.d.
n.d.
*
*
Vehicle
MD-EX
MD-GA
RTS
RTS-MD-EX
RTS-MD-GA

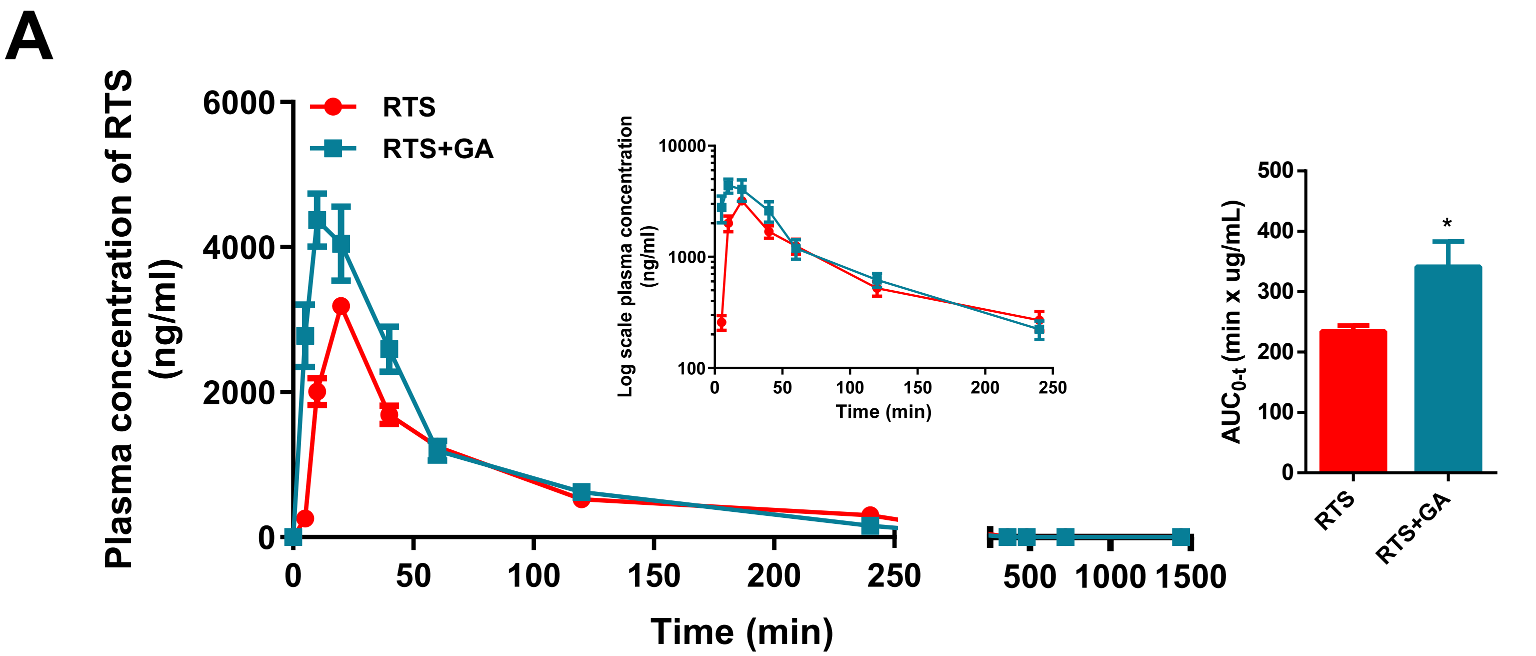

Pharmacokinetic parameters of RTS in rats

Groups	RTS	GA+RTS
T_{max} (min)	20.00 ± 0.00	13.33 ± 4.71
C_{max} (ng/mL)	3188.07 ± 144.65	4806.31 ± 133.74***
$AUC_{0\to1440}$ (min×μg/mL)	234.49 ± 34.07	341.52 ± 7.57*
V_Z/F (mL)	4058.24 ± 703.60	3439.4 ± 972.74
Cl/F (mL/min)	28.18 ± 0.92	19.53 ± 2.07**
$T_{1/2}$ (min)	100.24 ± 19.65	119.91 ± 20.39

Data were presented as mean ± S.D., n=3. *$p < 0.05$, **$p < 0.01$, ***$p < 0.001$ compared with the RTS groups.
$AUC_{0\text{-}1440}$: the area under the concentration vs time curve from 0 to 1440 min (the last time point tested).

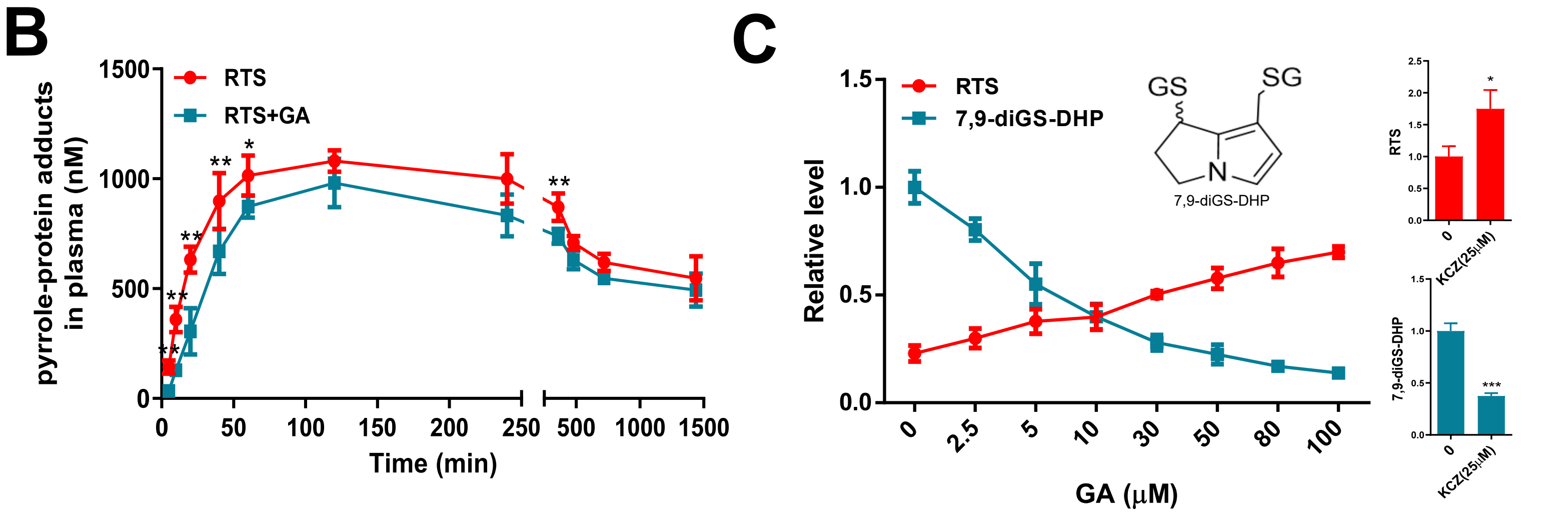

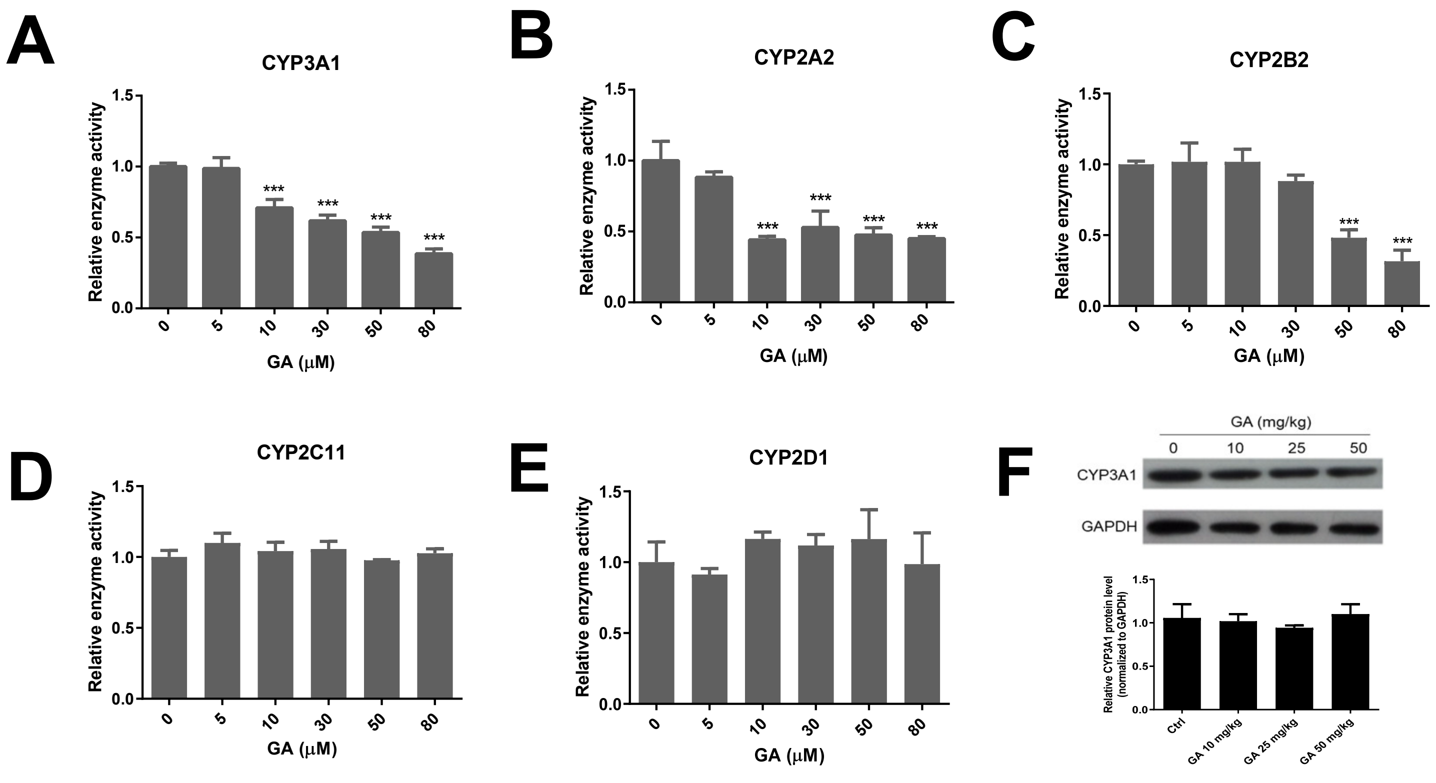
A
CYP3A1
B
CYP2A2
C
CYP2B2
D
CYP2C11
E
CYP2D1
F
Relative enzyme activity
GA (μM)
0
5
10
30
50
80
***
GA (mg/kg)
CYP3A1
GAPDH
Relative CYP3A1 protein level (normalized to GAPDH)
Ctrl
GA 10 mg/kg
GA 25 mg/kg
GA 50 mg/kg

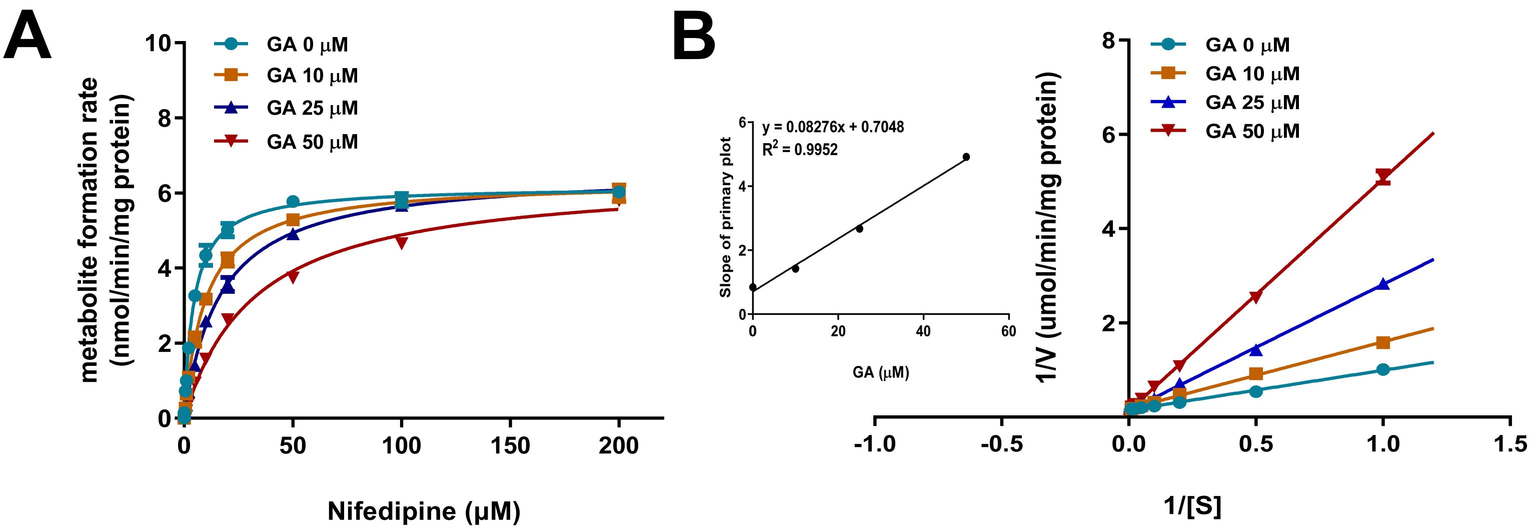

C

Kinetic parameters of Michaelis-Menten plots

GA (μM)	Vmax (nmol/min/mg protein)	Km (μM)	R^2
0	6.175±0.077	4.490±0.249	0.9925
10	6.326±0.079	9.922±0.484**	0.9947
25	6.586±0.083	16.74±0.735***	0.9961
50	6.473±0.146	32.61±2.204***	0.9921

Parameters of Lineweaver-Burk plot of each group

GA (μM)	Slope	Y-intercept when X=0.0	R^2
0	0.843±0.032	0.150±0.014	0.9729
10	1.424±0.040***	0.174±0.017	0.9850
25	2.671±0.041***	0.144±0.018	0.9955
50	4.916±0.064***	0.137±0.028	0.9968
