# Supplementary material for: Liquorice Extract and 18β-Glycyrrhetinic Acid Protect Against Experimental Pyrrolizidine Alkaloid-Induced Hepatotoxicity in Rats Through Inhibiting Cytochrome P450-Mediated Metabolic Activation
Source: Front Pharmacol. 2022 Mar 16;13:850859. doi: 10.3389/fphar.2022.850859 (PMC8966664; doi:10.3389/fphar.2022.850859)
Supplement: Supplementary file 2 [file DataSheet1.docx]

***Supplementary Materials***

**Liquorice Extract and 18β-Glycyrrhetinic Acid Protect against Pyrrolizidine Alkaloid-Induced Hepatotoxicity through Inhibiting Cytochrome P450-Mediated metabolic activation**

Zhangting Wang^1^, Jiang Ma^1^, Sheng Yao^2^, Yishen He^1^, Kai-Kei Miu^1^, Qingsu Xia^3^, Peter P. Fu^3^, Yang Ye^2^, Ge Lin^1#^

^1^ School of Biomedical Sciences, Faculty of Medicine, The Chinese University of Hong Kong, Hong Kong SAR, China

^2^ State Key Laboratory of Drug Research and Natural Products Chemistry Department, Shanghai Institute of Materia Medica, Chinese Academy of Sciences, Shanghai 201203, China

^3^National Center for Toxicological Research, U.S. Food and Drug Administration, Jefferson, Arkansas 72079, United States

**Running title:** GA attenuate Pyrrolizidine Alkaloids-Induced Hepatotoxicity

**Correspondence:** Professor Ge Lin,

School of Biomedical Sciences, Faculty of Medicine, The Chinese University of Hong Kong, Hong Kong SAR, China. E-mail: [linge@cuhk.edu.hk](mailto:linge@cuhk.edu.hk); Phone: (852) 3943 6824

**
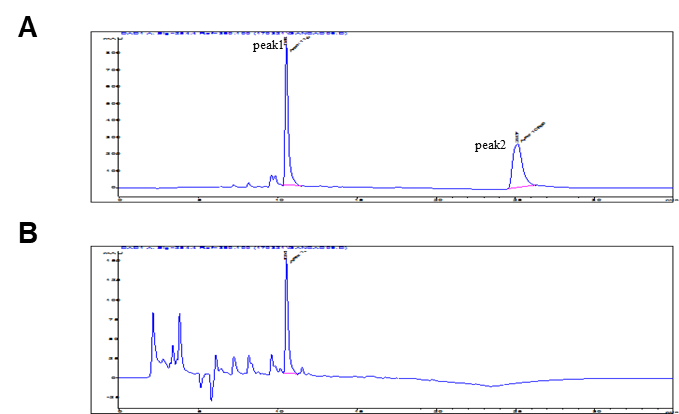
**

**Supplementary Figure S1.** HPLC-UV analysis of glycyrrhizin and GA in EX. (A) Representative chromatogram of standard glycyrrhizin (1.25 mg/mL, peak 1) and GA (0.625 mg/mL, peak 2) in a mixed solution. (B) Representative chromatogram of EX (5 mg/mL).


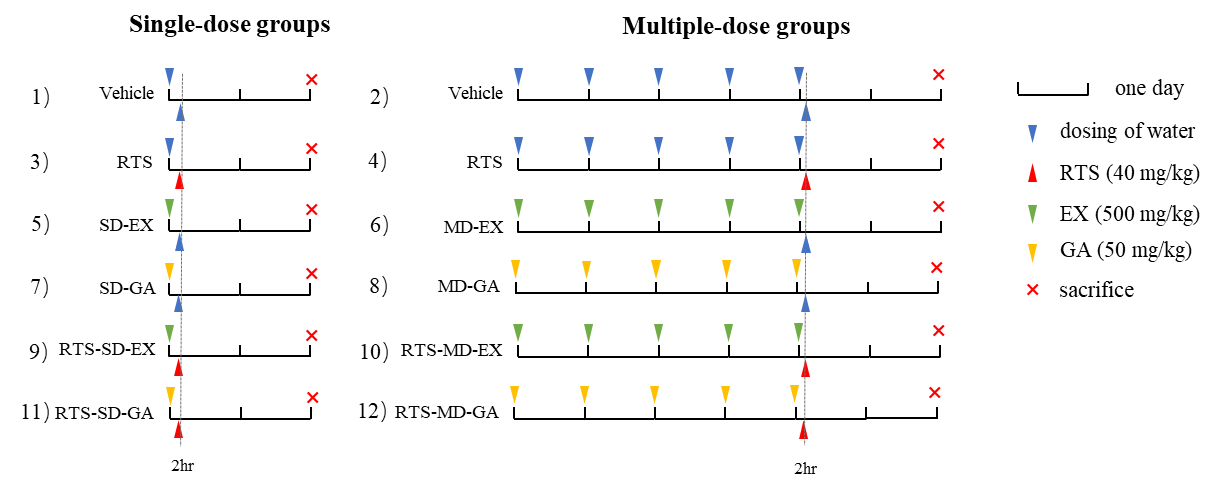


**Supplementary Figure S2.** Dosage regimens for 12 groups. In groups 9, 10 and 11, 12, EX or GA were considered as co-treatment with RTS, although RTS was given at 2 h after EX or GA dosing for the avoidance of solubility and large volume gavage problems in case of EX/GA and RTS dissolved together in the vehicle.

**
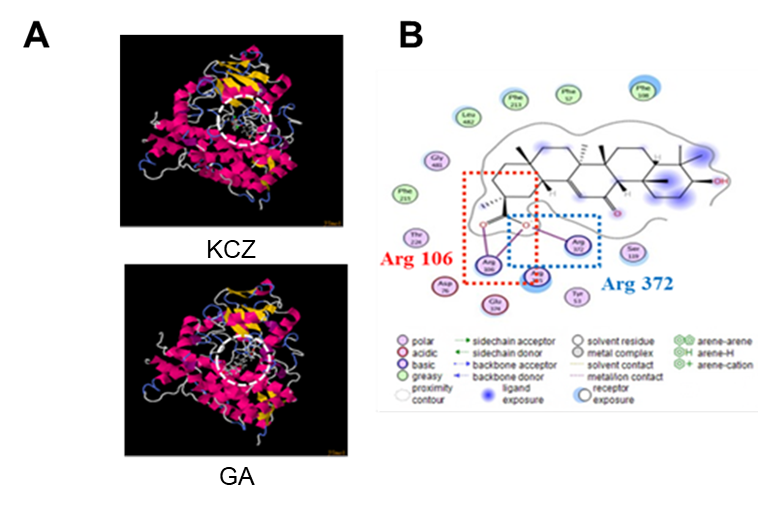
**

**Supplementary Figure S3.** Molecular docking of GA to human CYP3A4. (A) Three-dimensional diagram depicting predicted interaction of and KCZ and GA towards CYP3A4 binding domain. (B) Two-dimensional diagram displayed the docking model of GA in active site of human CYP3A4.

**Supplementary Table Ss1** Parameters for the “cocktail” CYP substrates and their corresponding metabolites.

| CYP enzyme | Compound | MRM transition | Frag.  (V) | CE  (V) | Dwell time (ms) | Km (µM) | Calibration curves^a^ | R^2^ |
| --- | --- | --- | --- | --- | --- | --- | --- | --- |
|  |  |  |  |  |  |  |  |  |
| 3A1/2 | Nifedipine | m/z 347.0-315.0 | 76 | 5 | 20 | 5.1-47 | Y=302022x+444755 | 0.991 |
|  | Oxidized nifedipine | m/z 345.0-284.1 | 158 | 29 | 20 |  | Y=2587093x+1648668 | 0.993 |
| 1A2 | Phenacetin | m/z 180.0-138.0 | 117 | 13 | 20 | 1.7-152 | Y=395887x+652944 | 0.990 |
|  | Acetaminophen | m/z 152.0-110.1 | 106 | 13 | 20 |  | Y=234838x+276627 | 0.990 |
| 2C11 | S-Mephenytoin | m/z 219.0-134.1 | 102 | 13 | 50 | 13-35 | Y=36968x+47623 | 0.994 |
|  | 4-hydroxymephenytoin | m/z 235.0-150.0 | 106 | 13 | 50 |  | Y=136703x+206500 | 0.990 |
| 2A2 | Coumarin | m/z 147.0-147.0 | 106 | 0 | 50 | 0.3-2.3 | Y=312106x+67818 | 0.999 |
|  | 7-hydroxycoumarin | m/z 162.9-162.9 | 143 | 0 | 80 |  | Y=181573x+516487 | 0.990 |
| 2B2 | Bupropion | m/z 240.0-184.0 | 91 | 9 | 20 | 67-168 | Y=3327719x+5193383 | 0.988 |
|  | Hydroxybupropion | m/z 256.0-238.0 | 81 | 9 | 20 |  | Y=4853952x+9510676 | 0.985 |
| 2D1 | Dextromethorphan | m/z 272.1-215.1 | 123 | 25 | 20 | 0.4-8.5 | Y=31706273x+2613402 | 0.989 |
|  | dextrorphan | m/z 258.1-199.1 | 143 | 29 | 20 |  | Y=1428201x+648620 | 0.987 |

^a^Y represents MS response, x represents compound concentration. Frag., fragmentor; CE, collision energy; R^2^, coefficient.

**Supplementary Table S2** Operating conditions of MS detector used in MRM for detection.

| Compound | Precursor Ion | Product Ion | Fragmentor | Collision Energy |
| --- | --- | --- | --- | --- |
| 7,9-diGS-DHP | 732 | 308.1 | 154 | 22 |
| 7,9-diGS-DHP | 732 | 118.1 | 154 | 66 |
| RTS | 352 | 138 | 190 | 20 |
| RTS | 352 | 120 | 190 | 30 |
| Pyrrole-protein adducts | 341.2 | 252.2 | 185 | 33 |

**Supplementary Table SS3** Grades definition of the liver damage scoring system.

| **Scoring parameters** | **0** | **1 (mild)** | **2 (moderate)** | **3 (severe)** |
| --- | --- | --- | --- | --- |
| CV endothelial damage | Absent | Focal involvement of some but not the entire endothelial vessel lining | Involvement of the entire vessel lining in a minority of vessels | Involvement of the entire vessel lining in a majority of vessels |
| Subendothelial hemorrhage of CV | Absent | Some but a minority of CV involved, or involvement of most of the CV without centrilobular extension | Majority of CV involved, with centrilobular extension in some but a minority of CV | The majority of CV involved, with centrilobular extension in a majority of CV |
| Sinusoidal hemorrhage of CV | Absent | Focal sinusoidal involvement of the centrilobular region, or involvement of most of the centrilobular region without extension into the mid-lobular region | Majority of the centrilobular region involved, with extension into the mid-lobular region in some but a minority of lobules | Majority of centrilobular region involved with extension into the mid-lobular region in a majority of lobules |
| Sinusoidal dilatation | Absent | Visible sinusoidal space | Early dilation around CV | Generalised dilatation around CV (Medusa head) |
| Coagulative necrosis of hepatocytes | Absent | Centrilobular involvement in some but not all lobules, or in the majority of lobules without mid-lobular extension | Centrilobular involvement in a majority of lobules, with mid-lobular extension in a minority | Centrilobular involvement in the majority of lobules, with mid-lobular extension in a majority |
| Apoptotic bodies | Absent | 1 – 2 | 3 - 5 | > 5 |
| Subendothelial fibrosis of CV | Absent | ≤ 50% luminal occlusion in most CV, or > 50% occlusion in a minority of CV | > 50% luminal occlusion without total luminal occlusion in a majority of CV, or total luminal occlusion in a minority CV | Total luminal occlusion in the majority of CV |
| Sinusoid fibrosis | Absent | Focal sinusoidal involvement of the centrilobular region, or involvement of most of the centrilobular region without extension into the mid-lobular region | Majority of the centrilobular region involved, with extension into the mid-lobular region in some but a minority of lobules | Majority of centrilobular region involved with extension into the mid-lobular region in a majority of lobules |

**Supplementary Table S4** Pharmacokinetic parameters of plasma pyrrole-protein adducts in rats.

| Groups | RTS | GA+RTS |
| --- | --- | --- |
| T_max_ (min) | 140.00 ± 91.65 | 160.00 ± 69.28 |
| C_max_ (nM) | 1112.81 ± 23.02 | 1093.38 ± 89.42 |
| AUC_0-1440 min_ (min×µM) | 1022.49 ± 25.04 | 856.49 ± 11.05 *** |

AUC_0-t_: the area under the concentration vs time curve from 0 to 1440 min (the last time point tested).

Data are expressed as mean ±SD (n=3). ****p* < 0.001 comparing with RTS group.
